# In Situ Cocktail Nanovaccine for Cancer Immunotherapy

**DOI:** 10.1002/advs.202207697

**Published:** 2023-09-22

**Authors:** Mohan Liu, Daoyuan Xie, Die Hu, Rui Zhang, Yusi Wang, Lin Tang, Bailing Zhou, Binyan Zhao, Li Yang

**Affiliations:** ^1^ Department of Biotherapy Cancer Center and State Key Laboratory of Biotherapy West China Hospital Sichuan University Chengdu 610041 China

**Keywords:** combination immunotherapy, in situ, nanovaccine, anti‐PD‐1, tumor microenvironment

## Abstract

In situ vaccination is a desirable strategy for cancer immunotherapy due to its convenience and capacity to target tumor antigens. Here, an in situ nanovaccine based on a cationic peptide with cholesterol‐modified, DP7‐C, for cancer immunotherapy is rationally designed, and developed a cancer nanovaccine that is easy to preparate. The nanovaccine includes cocktail small interfering RNAs (siRNAs) and immunologic adjuvant CpG ODNs, has synergistic effect in the cancer treatment. This nanovaccine can induce tumor cell death, promote antigen presentation and relieve immune suppression in the tumor microenvironment (TME). Moreover, this nanovaccine is administered to CT26 (hot) and B16F10 (cold) tumor model mice, in which it targeted the primary tumors and induced systemic antitumor immunity to inhibit metastasis. It is validated that the nanovaccine can convert cold tumors into hot tumors. Furthermore, the nanovaccine increased the immune response to anti‐PD‐1 therapy by modulating the TME in both CT26‐ and B16F10‐tumor‐bearing mice. The siRNA cocktail/CpG ODN/self‐assembling peptide nanovaccine is a simple and universal tool that can effectively generate specific tumor cell antigens and can be combined with immuno‐oncology agents to enhance antitumor immune activity. The versatile methodology provides an alternative approach for developing cancer nanovaccines.

## Graphical abstract

An in situ nanovaccine based on a cationic peptide with cholesterol‐modified, DP7‐C, for cancer immunotherapy is rationally designed, and develop a cancer nanovaccine that is easy to preparate. The nanovaccine includes cocktail small interfering RNAs (siRNAs) and immunologic adjuvant CpG ODNs, has synergistic effect in the cancer treatment. This versatile methodology provides an alternative approach for developing cancer nanovaccines.

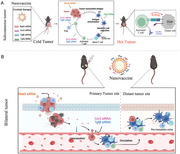

## 1 Introduction

Cancer has become the second leading cause of death worldwide after cardiovascular diseases.^[^ ^1^ ^]^ Traditional therapies mainly include surgery, chemotherapy, and radiotherapy.^[^ ^2^, ^3^ ^]^ Although cancer immunotherapy has enhanced cancer treatment outcomes, the lack of specific tumor antigen targets and the negative impact of the inhibitive tumor microenvironment (TME) on effector T cells are key barriers in immunotherapy.^[^ ^4^, ^5^, ^6^, ^7^, ^8^ ^]^ There are a variety of preclinical and clinical drugs that attempt to elicit tumor‐specific immune responses and remodel the immunosuppressive TME, such as monoclonal antibodies,^[^ ^9^ ^]^ small molecule inhibitors,^[^ ^10^ ^]^ and oncolytic viruses.^[^ ^11^, ^12^ ^]^ In addition, the therapeutic effect of agents targeting a single pathway or a single target is not favorable.^[^ ^13^ ^]^ Recently, researchers have focused on combining immunotherapy with other therapy types, including combining immune checkpoint inhibitors with conventional treatment methods (such as radiotherapy, chemotherapy, targeted drugs, and oncolytic virus therapy).^[^ ^14^, ^15^, ^16^ ^]^ In addition, combinations of multiple immune checkpoint inhibitors have been tested, and the combined use of agents with multiple targets has been explored preclinically.^[^ ^17^, ^18^, ^19^ ^]^ However, the ideal combination(s) that can induce synergistic effects to battle cancer remain to be identified. Therefore, it is necessary to explore rationally designed cancer vaccines to maximize antitumor effects.

Small interfering RNA (siRNA)‐based therapeutics are promising avenues for cancer treatment,^[^ ^19^, ^20^ ^]^ facilitating the simultaneous silencing of multiple target genes in vivo. However, the translation of clinical therapies is impeded by the lack of strategies that ensure safe, effective, and spatially and temporally controllable delivery of nucleic acids and that induce innate immune responses.^[^ ^21^, ^22^ ^]^ Furthermore, the unfavorable physicochemical characteristics of siRNA largely prevent its diffusion across cellular membranes, impeding its ability to reach the cytoplasm, where it can engage the RNAi machinery.^[^ ^23^ ^]^ The clinical translation of siRNA therapeutics has therefore been dependent on chemical modification and the development of sophisticated delivery platforms to improve stability, limit immune activation, facilitate internalization, and increase target affinity.^[^ ^23^, ^24^ ^]^ To overcome the challenges, siRNA delivery technology has greatly advanced in the past two decades.^[^ ^24^ ^]^ Nanotechnology‐based delivery systems, for example, include a variety of materials and targeting modalities to overcome challenges in RNAi therapy.^[^ ^24^, ^25^ ^]^ Naked siRNA itself may be a poor immunogen, but siRNA conjugated to peptides, polymers (i.e., through PEGylation), or antibodies can be immunogenic.^[^ ^26^ ^]^ Furthermore, nanoparticles serve as a protective delivery system for siRNA, increasing the stability and solubility of drugs and extending their half‐life.^[^ ^23^, ^27^ ^]^

The TME is an intricate network containing various cells and factors that interact with tumor cells.^[^ ^28^, ^29^ ^]^ Thus, the TME significantly influences therapeutic response and clinical outcome.^[^ ^30^ ^]^ Microenvironment‐targeted therapy strategies include releasing tumor antigens, suppressing tumor angiogenesis, activating and recruiting immune effector cells in the inhibitory tumor microenvironment and controlling soluble immune suppressive factors. Although a number of efforts have been devoted to targeting TME components to achieve therapeutic benefits,^[^ ^26^ ^]^ many clinical trials targeting the TME have regrettably failed to show exciting results in cancer patients.^[^ ^31^, ^32^ ^]^ In addition, understanding the fundamental mechanisms of the TME in depth, developing reliable biomarkers to guide TME‐targeted therapy, and applying combinatory therapy are promising aims for improving the response to immunotherapy.^[^ ^26^, ^33^ ^]^ The development of rational drug combinations that can simultaneously target tumor cells and the microenvironment may represent a solution to overcome therapeutic barriers.

Herein, we report a nanovaccine designed to remodel the immunosuppressive TME that plays a role as an in situ vaccination for effective synergistic cancer therapy. DP7‐C, a low‐toxicity cholesterol‐modified form of the antimicrobial peptide (AMP) DP7, has been demonstrated to act not only as a satisfactory nanocarrier but also as an adjuvant that directly interacts with immune cells.^[^ ^34^, ^35^ ^]^ Based on clinical database analysis and differential gene expression analysis of RNA‐seq data to determine the differences between ‘hot’ and ‘cold’ model mice, we established a nanovaccine carrying multiple elements:^[^ ^1^ ^]^ STAT3 siRNA to induce tumor cell apoptosis and the release of tumor‐specific antigens;^[^ ^2^ ^]^ CCR2 siRNA to relieve the inhibition of macrophages and/or myeloid‐derived suppressor cells (MDSCs);^[^ ^3^ ^]^ TGF‐β siRNA to eliminate immune cell‐based immunosuppression in the TME; and^[^ ^4^ ^]^ the TLR9 agonist CpG ODNs to activate infiltrating antigen‐presenting cells and recruit intratumoral dendritic cells (DCs) to cross‐present tumor antigens. This cocktail vaccine produces effective specific immunity against tumor cells and enhances antitumor immune activity. As expected, this nanovaccine exhibited a synergistic antitumor effect compared with single‐target therapy in vivo and in vitro. Our nanovaccine changed the immunosuppressive TME into an effector T‐cell‐favorable environment in both CT26 (hot) and B16F10 (cold) mice. Furthermore, our nanovaccine induced a tumor‐specific immune response to prevent tumor recurrence and metastasis. Notably, administering both an anti‐PD1 antibody (αPD‐1) and the nanovaccine achieved synergistic antitumor effect, reducing the proportion of regulatory T (Treg) cells and increasing the numbers of CD4^+^ and CD8^+^ T cells. Compared with virus‐based cancer therapies, cationic peptides have promising safety advantages and ease of production on a large scale. Compared with other nonviral‐based in situ vaccination, our strategy has the synergetic advantage of inducing antigen release and capture and effector immune cell activation and thus has better therapeutic effects. These data strongly suggest the utility of our new strategy for the development of nanotherapy‐based combination cancer immunotherapies.

## 2 Results and Discussion

### 2.1 Different Gene Signatures are Associated with the Immune State of Tumors

**Figure 1:**
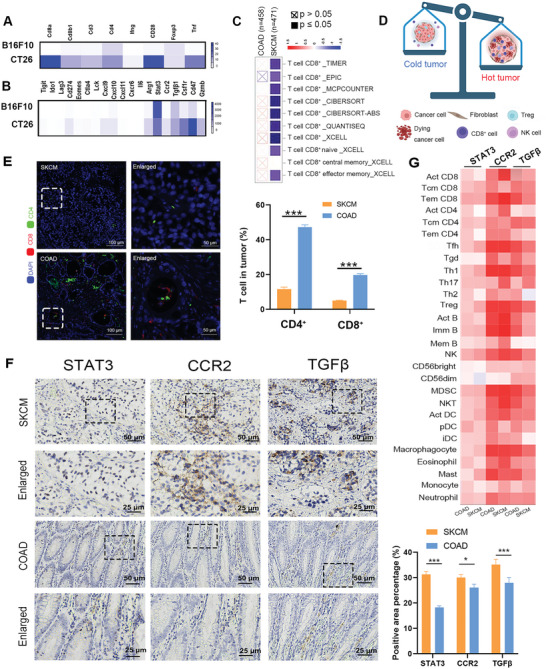
The CT26 colorectal carcinoma model features an immune‐inflamed phenotype compared with the B16F10 melanoma model. A,B) RNA was isolated from CT26 (A) and B16F10 (B) tumors, and the immune‐related transcriptome of each tumor was analyzed. A heatmap of select genes is shown, with the data presented as fold change values. C) Significant differences in infiltrating T cells between COAD and SKCM patient samples. D) Schematic illustration of differences in immune cell infiltration in “cold” and “hot” tumors. E) Immunofluorescence staining to assess CD4^+^ and CD8^+^ T‐cell infiltration in COAD and SKCM patient samples. Results are shown as mean ± SD, *p < 0.05, **p< 0.01, ***p < 0.001. F) Immunohistochemistry staining for STAT3, CCR2, and TGFβ in COAD and SKCM patient samples. Results are shown as mean ± SD, *p < 0.05, **p< 0.01, ***p < 0.001. G) Correlations between the abundances of tumor‐infiltrating lymphocytes and the expression of target genes.

Previous studies reported that immunologically inflamed “hot” TMEs are associated with an antitumor CD8^+^ T‐cell response signature and better clinical responses to immune checkpoint blockade (ICB), whereas immunologically “cold” TMEs are associated with poor clinical responses to ICB.^[^ ^36^, ^37^, ^38^, ^39^ ^]^ To explore the differences in infiltrating immune cells and gene expression between “cold” and “hot” TMEs, we constructed CT26 (colon cancer cells)‐ and B16F10 (melanoma cell)‐bearing mouse models. Subsequently, RNA‐seq revealed that the CT26‐bearing mice had higher expression of genes related to effector T cells, costimulatory molecules, and inflammatory cytokines than the B16F10‐bearing mice (**Figure** **1**A,B). To determine whether the differential response in colon adenocarcinoma (COAD) and skin cutaneous melanoma (SKCM) may be associated with differences in infiltrating immune cells, we performed bioinformatics analysis (http://timer.cistrome.org/; http://cis.hku.hk/TISIDB/), and the results suggested that the tumor immune microenvironment in COAD is more inflamed and has greater immune infiltration than that in SKCM (Figure 1C,D). Moreover, immunofluorescence microscopy was performed on patient samples, and more CD4^+^ T and CD8^+^ T cells were present in the COAD patient samples than in the SKCM patient samples (Figure 1E). Furthermore, we identified TME‐associated DEGs between hot and cold tumors. Of these DEGs, STAT3, which causes tumor cell death that results in the release of tumor‐associated antigens (TAAs),^[^ ^40^ ^]^ CCR2 and TGF‐β attracted our attention because they play an important role in cell proliferation, invasion, and anti‐apoptosis in numerous cancers and suppress the immune response;^[^ ^41^, ^42^ ^]^ thus, they were selected as candidate targets. Furthermore, immunohistochemistry demonstrated that STAT3, CCR2 and TGF‐β were highly expressed in COAD and SKCM patient samples (Figure 1F). In addition, bioinformatics analysis showed that the expression levels of STAT3, CCR2 and TGF‐β were highly correlated with the degree of immune cell infiltration (Figure 1G). Intriguingly, novel immunotherapies targeting STAT3, CCR2 and TGF‐β have been studied.^[^ ^43^, ^44^, ^45^, ^46^, ^47^, ^48^ ^]^ However, no study has evaluated whether strategies simultaneously targeting these three points can synergistically inhibit the proliferation of cancer cells and induce TME reprogramming. To enhance the immune response, we added the Toll‐like receptor 9 agonist CpG‐ODNs as an immunologic adjuvant in the treatment regimen. We rationally designed an in situ nanovaccine containing the self‐assembling peptide DP7‐C, STAT3 siRNA (to induce tumor cell apoptosis), CCR2 siRNA and TGF‐β siRNA (to relieve immune suppression in the TME), and CpG‐ODNs (to stimulate DC maturation) to enhance the antitumor immune response. This novel design provides a mechanism for overcoming the resistance to ICB therapy caused by the inadequate cancer antigen presentation and lack of cancer‐specific CD8^+^ T cells in the TME.

### 2.2 Characterization of DP7‐C/siRNA and DP7‐C/CpG ODN Nanoparticles

**Figure 2:**
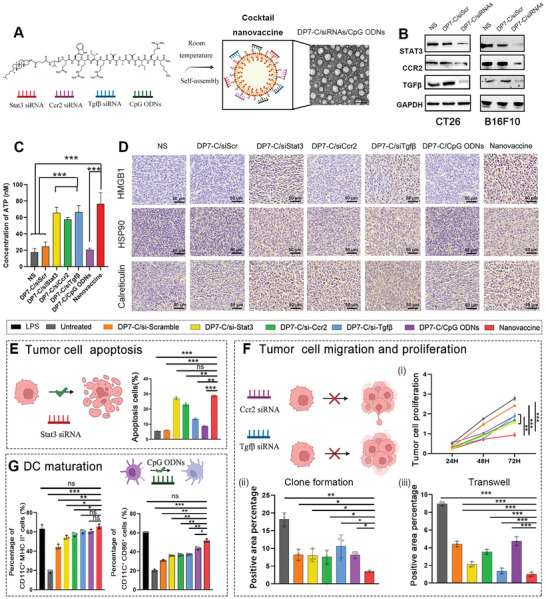
The nanovaccine induces combined antitumor effects and TME reprograming in vitro. A) Schematic illustrating the structure of the nanovaccine. B) Target gene expression at the protein level was detected by Western blot. C) Quantification of ATP levels in nanovaccine‐treated CT26 cells. D) In vivo immunogenic cell death induction upon nanovaccine treatment in CT26‐bearing mice. E) The percentage of apoptotic tumor cells in groups treated with DP7‐C, DP7‐C/si‐scramble, DP7‐C/siStat3, DP7‐C/siCcr2, DP7‐C/siTgfβ, DP7‐C/CpG ODNs or the nanovaccine for 48 h. F) Cell proliferation in groups treated with DP7‐C/si‐scramble, DP7‐C/siStat3, DP7‐C/siCcr2, DP7‐C/siTgfβ, DP7‐C/CpG ODNs or the nanovaccine for 48 h was measured by CCK8 (i), clone formation assays (ii) and transwell assay (iii). G) The percentage of CD11c^+^MHCII^+^ and mature BMDCs in groups treated with DP7‐C/si‐scramble, DP7‐C/siStat3, DP7‐C/siCcr2, DP7‐C/siTgfβ, DP7‐C/CpG ODNs or the nanovaccine. (Results are shown as mean ± SD, *p < 0.05, **p< 0.01, ***p < 0.001)

siRNA shows excellent pharmaceutical prospects in treating cancers.^[^ ^45^ ^]^ In this study, siRNA was used to silence target gene expression, and DP7‐C were used as the delivery system for our in situ vaccine (**Figure** **2**A). Gel retardation experiments showed that the optimal prepared mass ratios of DP7‐C to siRNA and DP7‐C to CpG ODNs were 5:1 (Figure S1A, Supporting Information). The particle size distribution of DP7‐C was ≈36.08 ± 2.792 nm (polydispersity index = 0.051) (Figure S1B, Supporting Information), and the zeta potential of DP7‐C was ≈36.8 ± 6.82 Mv (Figure S1C, Supporting Information). After incubating DP7‐C with the siRNA (polydispersity index = 0.076) or CpG ODNs (polydispersity index = 0.064), the particle size was larger than that of DP7‐C alone, while the zeta potential was lower than that of DP7‐C alone, indicating that DP7‐C can form complexes with the siRNA or CpG ODNs (Figure S1B,C, Supporting Information). The TEM image of DP7‐C/siRNAs/CpG ODNs showed a spherical morphology with an average diameter of 54.30 ± 6.81 nm, in line with its particle size (Figure 2A). To investigate the ability of DP7‐C to protect siRNA from RNase degradation, we treated the DP7‐C/siRNA complex with RNase A to mimic the enzymatic environment. After treating the DP7‐C/siRNA complex with RNase A for 0, 0.5, 1, 2, and 4 h, the DP7‐C/siRNA complex showed no significant change, while naked siRNA was completely degraded after RNase A treatment for 0.5 h under the same conditions (Figure S1D, Supporting Information), indicating that DP7‐C can protect siRNA from RNase degradation. In addition, the storage stability of DP7‐C/siRNA was focused. To confirm the long‐term storage of DP7‐C/siRNAs/CpG ODNs, the physicochemical properties for 4 weeks after storage at room temperature was tested. The results showed that naked siRNA obviously disappeared after 7 days, while DP7‐C/siRNA remained clear after 28 days (Figure S1E, Supporting Information). We then characterized particle size and zeta potential, and DP7‐C/siRNAs/CpG ODNs showed no particle size change and also maintained approximately zeta potential for at least 4 weeks after production (Figure S1F,G, Supporting Information). Encapsulation efficiency was measured by the RiboGreen assay and achieved more than 97% (Figure S1H, Supporting Information). No significant change in encapsulation efficiency of DP7‐C/siRNA stored for 4 weeks at room temperature (Figure S1H, Supporting Information). These results support the favorable stability of DP7‐C/siRNAs/CpG ODNs, which satisfied the current in‐use stability instructions of peptide‐based nanovaccine. Our findings suggest that DP7‐C can bind siRNA and CpG ODNs to efficiently form stable complexes, which suggests that DP7‐C has the potential as a small nucleic acid carrier.

### 2.3 Efficient In Vitro and In Vivo siRNA Delivery of DP7‐C

Considering that DP7‐C is a potential carrier for siRNA, we examined its delivery efficiency by cell transfection. B16F10 and CT26 cells were subjected to serum‐free in vitro transfection. Different amounts of Lipofectamine 2000, PEI25K, and DP7‐C were incubated in vitro with the same amount of Cy3‐siRNA according to the optimal mass ratios. After 24 h, the transfection efficiency of each group was analyzed by flow cytometry and fluorescence microscopy. The efficiency of siRNA transfection into CT26 and B16F10 cells via DP7‐C was nearly achieved 80% and better than that of Lipofectamine 2000, and no significant difference with PEI25K (Figure S2A, Supporting Information).

Successful escape from late endosomes/lysosomes is crucial for improved siRNA delivery efficiency. Extracellular substance uptake types mainly include macropinocytosis, caveolin‐mediated endocytosis, and clathrin‐mediated endocytosis.^[^ ^49^ ^]^ We used inhibitors of the three pathways in cultured cells and found that the uptake efficiency was significantly reduced after treatment with chlorpromazine, an inhibitor of the clathrin pathway (Figure S2B, Supporting Information). After internalization, DP7‐C/siRNA was sequentially transported through early endosomes, late endosomes, and lysosomes. To determine the intracellular localization, cells were treated with FAM‐siRNA or DP7‐C/FAM‐siRNA and then observed by confocal laser scanning microscopy (Figure S2C, Supporting Information). Upon FAM‐siRNA treatment, fluorescence was mainly observed in late endosomes and lysosomes, while a large amount of fluorescence delivered by DP7‐C was located in the cytoplasm, suggested that siRNA loaded by DP7‐C released from lysosomes and endosomes into the cytoplasm can play an effective role in the silencing of target genes (Figure S2C, Supporting Information). We also explored the toxicity of DP7‐C in vitro. Different concentrations of cationic liposomes were used to treat 293T cells for 24 h, and DP7‐C showed outstanding safety compared to Lipofectamine 2000 and PEI25K (Figure S3A,B, Supporting Information). These results indicated that siRNA encapsulated by DP7‐C has the ability to overcome delivery barriers, providing evidence for DP7‐C as an optimal siRNA carrier.

Furthermore, we have detected whether DP7‐C could deliver multiple siRNAs. First, we tested the transfection ability of DP7‐C to a single siRNA (FITC‐labeled), and the FITC^+^ siRNA was as high as 82.35% (Figure S4A, Supporting Information). Subsequently, we tested the transfection ability of DP7‐C to two different siRNAs (FITC‐labeled and Cy3‐labeled). The flow cytometry results showed that FITC^+^ cells account for 83.91% and Cy3^+^ cells account for 81.95%, meanwhile, Cy3 and FITC fluorescence were completely merged in cells (Figure S4A, Supporting Information). We further transfected three different siRNAs by DP7‐C (FITC‐labeled, Cy3‐labeled and Cy5‐labeled). Similarly, FITC^+^ cells account for 73.64%, Cy3^+^ cells account for 75.31% and Cy5^+^ cells account for 74.99%. As expected, the fluorescence of Cy3, Cy5 and FITC were completely overlapped (Figure S4A, Supporting Information). The above results suggested that DP7‐C had no differential selection for different siRNAs since DP7‐C as a cationic peptide binds siRNA through electrostatic interaction, and the transfection efficiency for different siRNA is as high as 73% respectively (Figure S4A, Supporting Information).

To confirm whether transfection of tumor cells with combination siRNAs via our DP7‐C/siRNAs delivery system would downregulate the expression of the target protein in the transfected cells. qPCR and western blotting analysis were performed. The mRNA and protein expression of targets was substantially decreased after transfection 48 h (Figure 2B; Figure S4B, Supporting Information), suggesting that the expression of immunosuppressive genes can be suppressed by this technology and confirming the efficacy of RNAi‐based silencing therapies in vitro. Moreover, the expression of the targets was not obviously altered by si‐scramble‐carrying DP7‐C compared with no treatment, indicating that the gene silencing effects were sequence‐specific (Figure 2B; Figure S4B, Supporting Information). Thus, we confirmed that the siRNA used in the current study successfully silenced target gene expression in vitro. On this basis, we provided proof of concept for generating cocktail siRNA formulations to be administered via the DP7‐C delivery system through rational combination of siRNAs targeting different genes regulating the TME.

### 2.4 Nanovaccine Induces Immunogenic Cell Death and Induces Synergistic Antitumor Effects and TME Reprograming In Vitro

Emerging evidence shows that the efficacy of anticancer drugs depends on their ability to induce immunogenic cell death (ICD).^[^ ^50^, ^51^, ^52^ ^]^ ICD produces neoantigens and improves the T‐cell response against different tumors, indicating that ICD can enhance the antitumor immunity elicited by αPD‐1.^[^ ^51^ ^]^ We investigated whether the siRNA cocktail/CpG ODN/self‐assembling peptide formulation elicited ICD when administered as an in situ nanovaccine in vivo and in vitro. The immunogenic effects of ICD are mainly mediated by damage‐associated molecular patterns (DAMPs), including translocated calreticulin (CRT), secreted ATP and released high mobility group protein B1 (HMGB1).^[^ ^48^, ^52^ ^]^ In order to identified whether DP7‐C/siRNAs/CpG ODNs could induce ICD, we measured DAMPs after tumor cells (CT26 and B16F10) were stimulated with DP7‐C/siRNAs/CpG ODNs in vitro. Tumor cells treated with DP7‐C/siRNAs/CpG ODNs secreted higher levels of ATP than untreated groups (Figure 2C; Figure S5A, Supporting Information). Furthermore, immunofluorescence staining showed that CRT was translocated into the cell membrane and the increased HMGB1 and HSP90 release were also visualized by DP7‐C/siRNAs/CpG ODNs (Figure S6A, Supporting Information). To further define DP7‐C/siRNAs/CpG ODNs induce ICD through specific components or as a whole, we evaluated the in vivo DAMPs of single DP7‐C/siRNA toward B16F10 and CT26 cells. Elevated secretion of ATP in the medium in tumor cells treated with DP7‐C/siStat3, DP7‐C/si‐Ccr2 and DP7‐C/si‐Tgfβ (Figure 2C; Figure S5A, Supporting Information). Meanwhile, CRT exposure on membrane was closely related to the DP7‐C/siStat3, DP7‐C/si‐Ccr2 and DP7‐C/si‐Tgfβrespectively, while DP7‐C/siRNAs/CpG ODNs was able to trigger more CRT exposed on the cell surface compared with the single DP7‐C/siRNA(Figure S6A, Supporting Information). Similarly, HMGB1 and HSP90 release became more dominant for the groups of DP7‐C/siRNAs/CpG ODNs than single siRNA treated groups (Figure S6A,B, Supporting Information). Subsequently, we analyzed whether DP7‐C/siRNAs/CpG ODNs‐induced cell death is also immunogenic in vivo. After administrated by DP7‐C/siRNA in tumor‐bearing mice, the ICD markers were evaluated in tumor sections through IHC. The CRT in groups of DP7‐C/siStat3, DP7‐C/si‐Ccr2, DP7‐C/si‐Tgfβ and DP7‐C/siRNAs/CpG ODNs was exposed on the cell membrane and the HMGB1 and HSP90 expression were significant increased (Figure 2D; Figure S5B, Supporting Information). Above results suggested that DP7‐C as a carrier and also an immune adjuvant,^[^ ^35^ ^]^ carry STAT3, CCR2 and TGFβ siRNA separately or their combination could be effective in inducing ICD. In summary, these results demonstrated that DP7‐C/siRNAs/CpG ODNs is a candidate therapeutic cancer nanovaccine that induces ICD.

Inhibition of cancer cell proliferation and promotion of cancer cell apoptosis are important indicators of antitumor effects. The nanovaccine also showed optimal apoptosis‐stimulating capacity (Figure 2E; Figure S7A, Supporting Information). Moreover, we detected the effects of the designed nanovaccine on the proliferation of cancer cells using the CCK8 assay and clone formation assay (Figure 2F). Compared with the untreated group, the groups treated with DP7‐C/siRNA showed significantly inhibited growth of cancer cells (Figure 2F; Figure S7B, Supporting Information). As expected, the group treated with the nanovaccine presented the most effective inhibition of proliferation (Figure 2F; Figure S7B, Supporting Information). Subsequently, tumor cell migration and tumor cell invasion were evaluated using a transwell assay. The mobility and invasion of cancer cells were suppressed by DP7‐C/siRNAs (Figure 2F; Figure S7C, Supporting Information). Moreover, DP7‐C/si‐scramble showed no impact on these cancer cell characteristics, implying specificity of the cocktail siRNAs. These experiments demonstrated that targeting single or multiple targets can result in antitumor effects by suppressing the proliferation, migration, and invasion of cancer cells. CRT acts as an “eat me” signal by binding to the surface maker CD91 on DCs, stimulating them to grow into matured DCs,^[^ ^52^ ^]^ which could induce adaptive immune responses by naive T cells by identifying tumor antigens and presenting them to T cells. Thus, we assessed the effect of the nanovaccine on DC maturation and used CD86 and CD80 as markers of mature DCs. Our analysis revealed that LPS (as a positive control) stimulated 53.21% DC maturation and the nanovaccine treatment more effectively promoted BMDC maturation (51.08%) than individual treatment (Figure 2G; Figure S7D,E, Supporting Information). MHC II^+^ is related to antigen processing,^[^ ^53^ ^]^ and the MHC II^+^ CD11c^+^ jumped to 61.6% in the nanovaccine compared to 19.75% in the untreated group (Figure 2G; Figure S7D,E, Supporting Information). Overall, the cocktail siRNAs delivered by DP7‐C to cancer cells exerted favorable antitumor effects.

In situ vaccination is an immunotherapy approach that directly injects immunostimulatory reagents to disrupt local immunosuppression and stimulate an effective immune response against the tumor.^[^ ^54^ ^]^ We used a rational approach to design a siRNA cocktail/CpG ODN/self‐assembling peptide nanovaccine that induces tumor cell apoptosis and the release of immunogenic DAMPs, which are natural immune adjuvants. We validated that our nanovaccine induces crucial ICD hallmarks, including translocation of CRT and increased HSP90, HMGB‐1, and ATP levels, suggesting that the nanovaccine successfully induces ICD to potentiate DC maturation, which may in turn enhance immune stimulatory or subvert immune suppressive effects through cytotoxic T lymphocytes induction for tumor elimination. Despite DP7‐C load one of si‐Stat3, si‐Ccr2, si‐Tgfβ could trigger ICD and exhibited enhanced the immunopotentiation effect, the cocktail vaccine seemed to exhibit the best ICD and antitumor effect. These results reveal that DP7‐C can be used to carry STAT3, CCR2 and TGFβ siRNA, and the combined vaccination was more effective in inducing ICD.

### 2.5 Potent Antitumor Effects of the Nanovaccine in Hot and Cold Tumor Models

**Figure 3:**
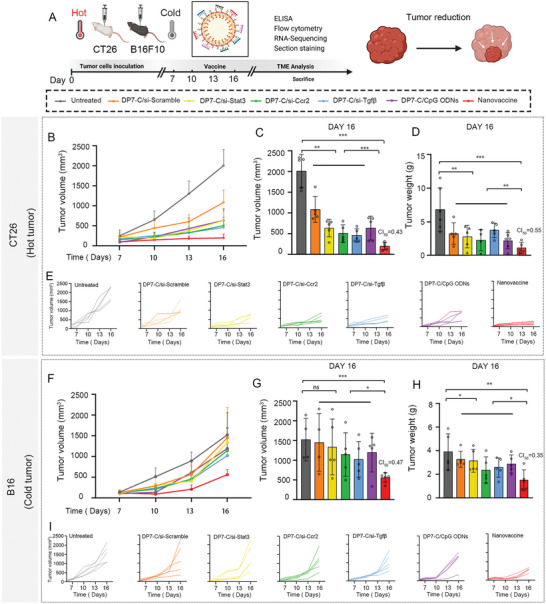
Nanovaccine treatment results in favorable therapeutic effects in various models. A) Schematic of the experimental workflow: subcutaneous injection of the nanovaccine into 8‐week‐old mice to study the in vivo therapeutic efficiency in an in situ tumor model. B–E) Antitumor effects of the nanovaccine in the CT26 in situ tumor model. B) Tumor volumes, C) mean tumor volumes on day 16, D) tumor weights, and E) tumor growth curves of the individual mice. F–I) Antitumor effects of the nanovaccine in the B16F10 in situ tumor model. F) Tumor volumes, G) mean tumor volumes on day 16, H) tumor weights, and I) tumor growth curves of the individual mice.

We hypothesized that CT26 and B16F10 tumor models would have positive responses to the cocktail vaccine because the included targets were selected according to their association with an immune‐suppressive TME. To verify our hypothesis, CT26 tumor‐bearing mice were treated with various combinations of si‐Stat3, si‐Ccr2, si‐Tgfβ, and CpG ODNs. Next, we compared the efficacy of the cocktail regimen with that of the cocktail regimen minus one or two components to assess the contribution of each component to the antitumor effect of the cocktail combination. As shown in the administration scheme in **Figure** **3**A, the body weights and tumor volumes were recorded every two days. First, downregulated expression of STAT3, CCR2, and TGFβ in tumor tissues was demonstrated by western blot, which showed that DP7‐C could effectively deliver the siRNAs to their targets inside cells following intravenous administration (Figure S8, Supporting Information). Then, we analyzed the antitumor effect after different treatments. All treatment groups exhibited partial tumor suppression compared with the untreated and DP7‐C/si‐scramble‐treated groups, while the group treated with the siRNA cocktail nanovaccine showed optimal tumor inhibition (Figure 3B–E). The tumor inhibition rate (based on tumor weight) in the nanovaccine group was 84.4%, and the combination index (CI) calculated with CompuSyn software was 0.55, indicating a synergetic effect of the four components (Figure 3C). Moreover, we compared the efficacy of the cocktail nanovaccine with that of each minus‐one component treatment modality to evaluate the contribution of each component to the antitumor effect of the combination treatment, the cocktail nanovaccine has minimum weight and encumbrance (Figure S9A–D, Supporting Information), further confirmed the synergetic effect of nanovaccine.

Likewise, the cocktail nanovaccine was applied to treat the B16F10 tumor model, which has less immune cell infiltration than the CT26 tumor model and regarded as a cold tumor model. The cocktail nanovaccine strategy (CI = 0.35) exerted a superior antitumor effect compared with the control and single modality treatments (Figure 3F–I). In addition, treatment with the cocktail nanovaccine regimen minus one treatment modality (DP7‐C/si‐Ccr2+CpG ODNs and DP7‐C/si‐Tgfβ+CpG ODNs) resulted in the inhibition of tumor growth compared with treatment with the control or single modality regimens(Figure S9G–J, Supporting Information). The other treatments had no distinct effects on B16F10 tumor growth. More importantly, unlike in the hot CT26 tumor model, in the B16F10 tumor model, none of the single modality treatments resulted in tumor regression (Figure 3F–I). On the other hand, the cocktail nanovaccine outperformed all the other treatments in controlling B16F10 tumor growth, suggesting that superior antitumor effects were achieved in the cold B16F10 model when the immune response was enhanced by a multifaceted immunotherapy combination (Figure 3F–I). Cold tumors, in this study represented by the B16F10 model, are more challenging to treat. The lack of T‐cell infiltration in cold tumor lesions may be due to several factors, such as a lack of tumor antigens, defects in antigen processing and presentation, a lack of T‐cell activation, and the inability of T cells to home to the tumor.^[^ ^55^ ^]^ Our experimental data indicate that the single treatments had no effect on B16F10 tumor growth. In contrast, treatment with a cocktail nanovaccine could address different treatment barriers related to TME. Each of the components of the cocktail nanovaccine was essential for controlling tumor growth, as the removal of one treatment modality resulted in diminished effectiveness of the combination.

Subsequently, TUNEL staining, and immunohistochemistry were performed to detect Ki‐67 and CD31. The results showed that the cocktail group had significantly altered cellular morphology and obvious cell apoptosis (Figure S10, Supporting Information), which further demonstrated the predominant inhibitory effect of the cocktail vaccine on tumor growth. The safety of the nanovaccine was assessed in experimental mice. Additionally, no obvious histological toxicity was observed following administration of the nanovaccine to mice (Figure S11, Supporting Information).

### 2.6 Cocktail Nanovaccine Remodels the Tumor Immune Microenvironment

**Figure 4:**
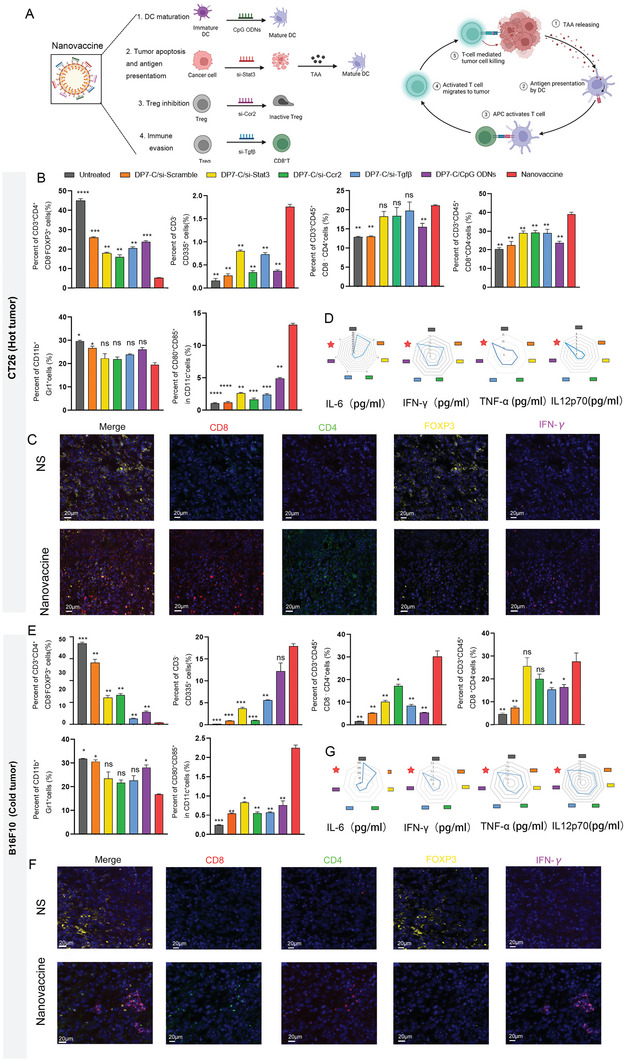
Administration of the nanovaccine promoted the immune response in vivo. A) Effect of the nanovaccine on the tumor microenvironment. B) Treatment with the nanovaccine induced robust antitumor immune cell responses in the CT26 tumor model. C) Immunostaining for immune markers in tumors from the untreated and nanovaccine‐treated groups in the CT26 tumor model. D) Tumor cytokine levels after treatment in the CT26 tumor model. E) Treatment with the nanovaccine induced robust antitumor immune cell responses in the B16F10 tumor model. F) Immunostaining for immune markers in tumors from the untreated and nanovaccine‐treated groups in the B16F10 tumor model. G) Tumor cytokine levels after treatment in the B16F10 tumor model.

Our cocktail nanovaccine was designed to induce tumor cells death by si‐Stat3 and release TAA initially. The CpG ODNs stimulate the antigen presenting function of DCs and TAA was delivered by mature DCs to T cells. As a result, a series of immunological responses are activated by si‐Ccr2 and si‐Tgfβ, including CD4^+^, CD8^+^T cell proliferation, cytotoxic cytokine secretion and Treg inhibition (**Figure** **4**A). Combined strategies for tumor killing, antigen presentation and immune infiltration enhancement, which are the necessary stages of cancer immunity cycle, have been reported to produce TAA and proliferate tumor‐specific T cells to propagate antitumor immunity (Figure 4A). To assess the infiltration of immune cells into the TME, we isolated tumors and evaluated the levels of tumor‐infiltrating cells by flow cytometry. In CT26 model, an enlarged population of NK, CD4^+^ T and CD8^+^ T cells was identified in tumors in all treatment groups compared with untreated groups. The single and minus‐one treatment elicited immune cells to traffic into tumors, and the population of immune cells was further expanded after combination therapy (Figure 4B; Figure S9E, Supporting Information). In addition, the Treg population was decreased by all therapies, as well as nanovaccine treatment was significantly restrained than that of other treatments. Moreover, we observed slight decreasing in the frequency of tumor‐infiltrating MDSCs in nanovaccine compared to untreated group, while non‐significant reduction could be detected for MDSC in the nanovaccine compared to the single modal therapies. Analyzing the frequency of DC cells, we found their elevation in mice injected with nanovaccine as compared with other groups. Moreover, the immune expression profiles of numerous markers were investigated by mIHC, which enables accurate identification of the immune contexture. Consistent with the results of flow cytometry, nanovaccine treatment afforded tumor infiltration of robust CD4^+^ and CD8^+^ T cells (Figure 4C), revealed that the ratio of CD4^+^ and CD8^+^ T cells to FOXP3^+^ cells were upregulated in tumor beds after combination treatment in CT26 tumor (Figure 4C). Meanwhile, the numbers of DCs upregulated and MDSCs down (Figures S12 and S13, Supporting Information). On the other hand, in B16F10 model, the treatment groups all have a higher number of CD8^+^ T cells and in total CD4^+^ T cells were increased compared to the control group, suggested that the activation of effector T cells (Figure 4E). Likewise, the single and cocktail regimen all expanded NK cells compared with the control (Figure 4E). In addition, an increased number of mature DCs was observed after treatment, demonstrated the boosting antigen presentation effect of nanovaccine (Figure 4E). It is well‐known that Treg cells and MDSC usually act an immunosuppressive factor in antitumor immunity. The decreased number of Treg cells and MDSCs in nanovaccine group were indicated that the reversing the immunosuppressive TME (Figure 4E). Differ from the CT26 model, the B16F10 tumor site was nearly non‐accessible to CD8^+^ T cells in the PBS group; however, in the nanovaccine‐treated group, the tumor phenotypes were restored to immune‐excluded types, allowing T cells to approach to tumor site (Figure 4E,F). The decrease in Treg cells and the increase in CD4^+^ and CD8^+^ T cells resulted in favorable CD4‐to‐Treg and CD8‐to‐Treg ratios in the cocktail treatment group. A high CD8‐to‐Treg ratio in melanoma has been correlated with a high objective response rate and long progression‐free survival after treatment.^[^ ^55^ ^]^ The image analysis also revealed that the number of DC cells infiltrated into tumor tissues was higher in the cocktail vaccine treatment group (Figure S12, Supporting Information). Immunosuppressive cells such as MDSCs, which is associated with poor prognosis and resistance to therapy, was reduced with the nanovaccine treatment (Figure S13, Supporting Information). These findings suggest that cocktail nanovaccines can generate effective antitumor T‐cell immune responses and modify the immune landscape to favor effector populations over immunosuppressive cell types. Furthermore, a decrease in serum proinflammatory cytokine IL6 and increase in IL12p70/TNF‐α/IFN‐γlevels was observed in the cocktail group (Figure 4D,G). IL‐12 is considered a potent agent to enhance antitumor immune response by activated of T lymphoblasts and NK.^[^ ^56^ ^]^ Meanwhile, IL12 induces IFN‐γ production, which as an inducer of mediators inhibiting anti‐tumor immune reactions.^[^ ^57^ ^]^ Notably, IL6/STAT3 signaling is activated in tumorigenesis and metastasis^[^ ^58^ ^]^ and IL‐6 plays an important role in MDSC generation.^[^ ^59^ ^]^ These data suggest that our nanovaccine achieving the desired antitumor effect in hot and cold tumors requires multifaceted immunotherapeutic combinations (Figure 4D,G). Improved tumor growth inhibition was accompanied by higher CD8^+^ T‐cell infiltration into the TME in the group treated with the cocktail nanovaccine than in the single‐regimen‐treated group. Taken together, these findings indicate that cocktail nanovaccine therapy can facilitate T‐cell infiltration and extend T‐cell activation, ultimately changing a cold TME to a hot one.

**Figure 5:**
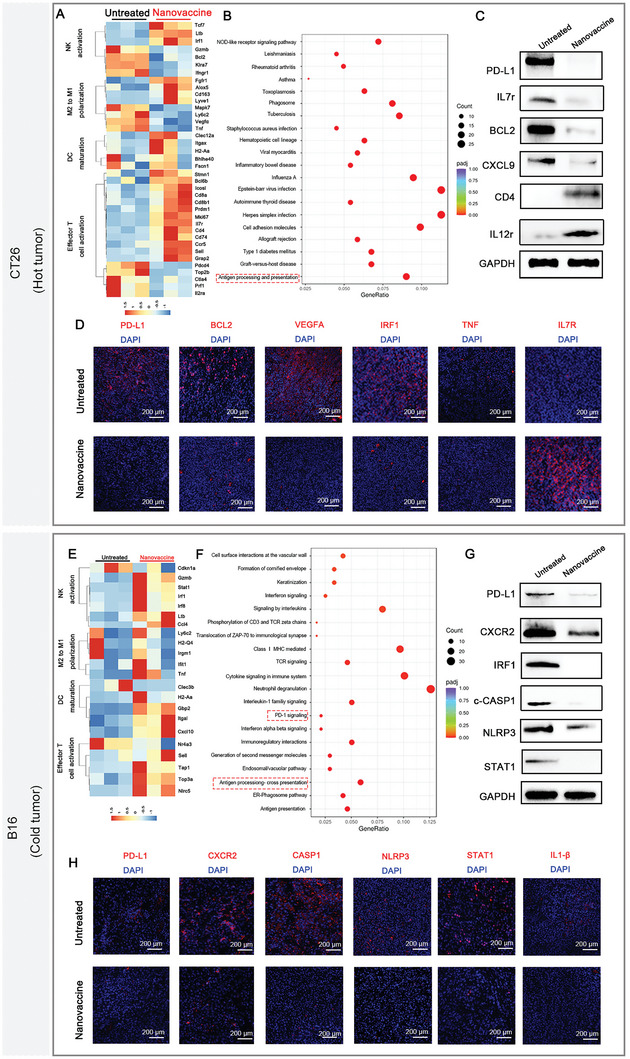
Characterization of the immune features of the microenvironment after nanovaccine treatment. A) Heatmap showing differentially enriched proteins between tumors of the untreated and nanovaccine‐treated groups in the CT26 tumor model. The altered proteins were mainly related to NK‐cell activation, M2 to M1 polarization, DC maturation and effector T‐cell activation. B) Bubble chart showing the enriched proteins related to antigen processing and presentation according to the GO analysis of nanovaccine‐treated tumors compared to untreated tumors in the CT26 tumor model. C) Western blot results confirming the altered protein expression identified by RNA‐seq analysis in the CT26 tumor model. D) Immunostaining results indicating the proteins with altered expression between tumors from the untreated and nanovaccine‐treated groups in the CT26 tumor model. E) Heatmap showing differentially enriched proteins between tumors from the untreated and nanovaccine‐treated groups in the B16F10 tumor model. The altered proteins were mainly related to NK‐cell activation, M2 to M1 polarization, DC maturation and effector T‐cell activation. F) Bubble chart showing the enriched proteins related to antigen processing and presentation and PD‐1 signaling according to the KEGG analysis of nanovaccine‐treated tumors compared to untreated tumors in the B16F10 tumor model. G) Western blotting results confirming the altered protein expression identified by RNA‐seq analysis in the B16F10 tumor model. H) Immunostaining results indicating the proteins with altered expression between tumors from the untreated and nanovaccine‐treated groups in the B16F10 tumor model.

To investigate the mechanism by which the nanovaccine inhibits tumor growth, we performed RNA‐seq to assess the immune response in the tumor tissue. As shown in **Figure** **5**, treatment with the nanovaccine had an observable impact on the expression of immune‐related genes compared with control treatment. RNA‐seq of the tumors revealed 1918 and 491 genes that were differentially expressed in treated mice compared to control mice in the CT26 model and B16F10 model, respectively (Figure 5A,E). On the other hand, nanovaccine treatment was correlated with increased expression of genes associated with T‐cell infiltration, NK‐cell activation, and DC maturation, supporting the flow cytometry data shown in Figure 4 (Figure 5B,F). In addition, decreased expression of genes involved in immunosuppression was observed in nanovaccine‐treated mice. Substantially reduced levels of *Cd274*, *Bcl2*, and *Vegfa* in nanovaccine‐treated mouse tumors in the CT26 model and significantly diminished levels of *Cxcr2* and *Stat1* in the nanovaccine‐treated mouse tumors in the B16F10 model were further confirmed by immunofluorescence staining (Figure 5C,D,E–G). Moreover, nanovaccine treatment was found to activate the inflammasome pathway and antigen processing and presentation, thus boosting antitumor immunity. The cocktail nanovaccine decreased tumor burden in the CT26 and B16F10 tumor models and was also associated with increased CD8^+^ T‐cell infiltration and proliferation and decreased Treg cell numbers in the tumor; furthermore, the recruitment of effector T cells into the TME was related to the increased expression of the chemokine ligand CXCL10 in the tumors. These results and the observation that the cocktail nanovaccine increased granzyme B and IFN‐γ production indicate that the nanovaccine converted B16F10 tumors into infiltrated‐inflamed phenotype tumors. These data suggest that the nanovaccine altered the tumor immune landscape, favoring an antitumor effector phenotype and decreasing immunosuppression.

### 2.7 Nanovaccine Reduced Postoperative Recurrence and Induced a Whole‐Body Antitumor Immune Response

**Figure 6:**
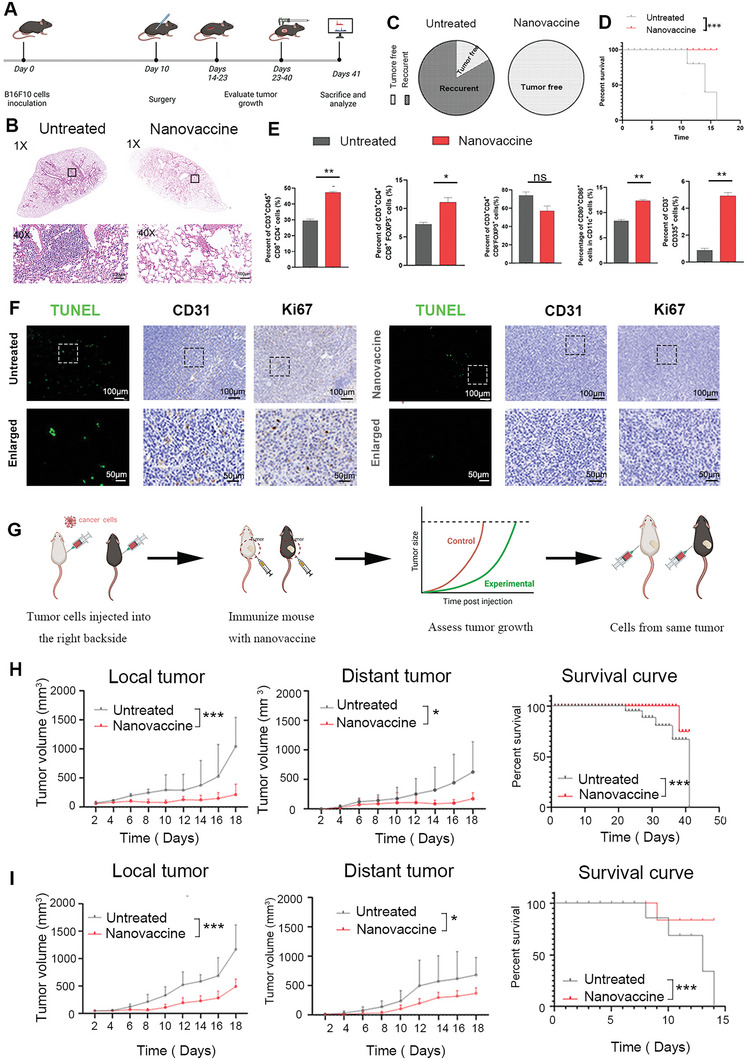
The nanovaccine inhibits postoperative recurrence of B16F10 tumors. A) Treatment schedule for postoperative immunotherapy. B) H&E staining of lungs from the untreated and nanovaccine‐treated groups after surgery with B16F10 tumor cells. C) Tumor‐free percentage of treatments for mice bearing recurrent B16F10 tumors. D) Overall survival time of mice bearing recurrent B16F10 tumors after nanovaccine treatment. E) Quantitative analysis of immune cells in tumor tissues. F) TUNEL, Ki67 and CD31 staining of B16F10 tumors excised after tumor recurrence. G–I) Effectiveness of in situ tumor vaccination in mice bearing bilateral syngeneic tumors. G) Schematic illustration of the in vivo immunotherapy studies. H,I) Tumor growth curves of the local tumors and distant tumors and survival profiles. H) Orthotopically implanted with CT26 cells, I) orthotopically implanted B16F10 cells.

Since the occurrence of tumor metastasis is an unavoidable and common problem in melanoma and accounts for low survival rates,^[^ ^60^, ^61^, ^62^ ^]^ we investigated whether our cocktail nanovaccine strategy could inhibit metastasis and generate systemic immunity against tumors. The antimetastatic effect was assessed using a postoperative B16F10 tumor model (**Figure** **6**A). The untreated group had apparent pulmonary metastasis, while the vaccinated group had no metastasis in any of the major organs (Figure 6B). Moreover, we observed that nanovaccine could significantly inhibit tumor recurrence with 4/5 tumor free compared with PBS (Figure 6C). The median survival time of mice that received the cocktail therapy was considerably prolonged (59 days) compared to that of the saline group (23 days) (Figure 6D). These results suggest that immunotherapy prevents tumor recurrence and prolongs survival time (Figure 6C,D). To explore the mechanism by which the cocktail nanovaccine prevents recurrence, immune cells were obtained from spleens for immunophenotypic analysis. The frequencies of mature DCs (CD80^+^CD86^+^) in lymph nodes in the vaccinated group were markedly higher than those in the untreated group, and the vaccinated group had more cytotoxic T‐cell infiltration (Figure 6E). The ability of nanovaccine to repress neovascularization, tumor cell proliferation and accelerate tumor apoptosis was demonstrated by CD31, KI67 and TUNEL staining in postoperative treatment (Figure 6F).

Considering its favorable effect on postoperative tumor recurrence, we wondered whether our cancer nanovaccine could be used to eradicate the primary tumor and tumors at distant sites. As shown in Figure 6G, we established bilateral orthotopic mouse colorectal (CT26) and melanoma (B16F10) tumor models. Seven days after tumor implantation, the nanovaccine was injected into the local tumor five times, each injection 3 days apart, while the distant tumor was left untreated. We found that our cancer vaccine controlled the size of both tumors (local and distant), and prolonged the survival of mice (Figure 6H,I). Our experiment suggests that local injection of the nanovaccine induces a systemic antitumor immune response. These results strongly suggest that our personalized nanovaccine is a profound tool that can be used to treat primary and metastatic tumors.

### 2.8 Nanovaccine Sensitizes Tumors to Anti‐Programmed Cell Death Protein 1 Therapy to Improve Efficacy

**Figure 7:**
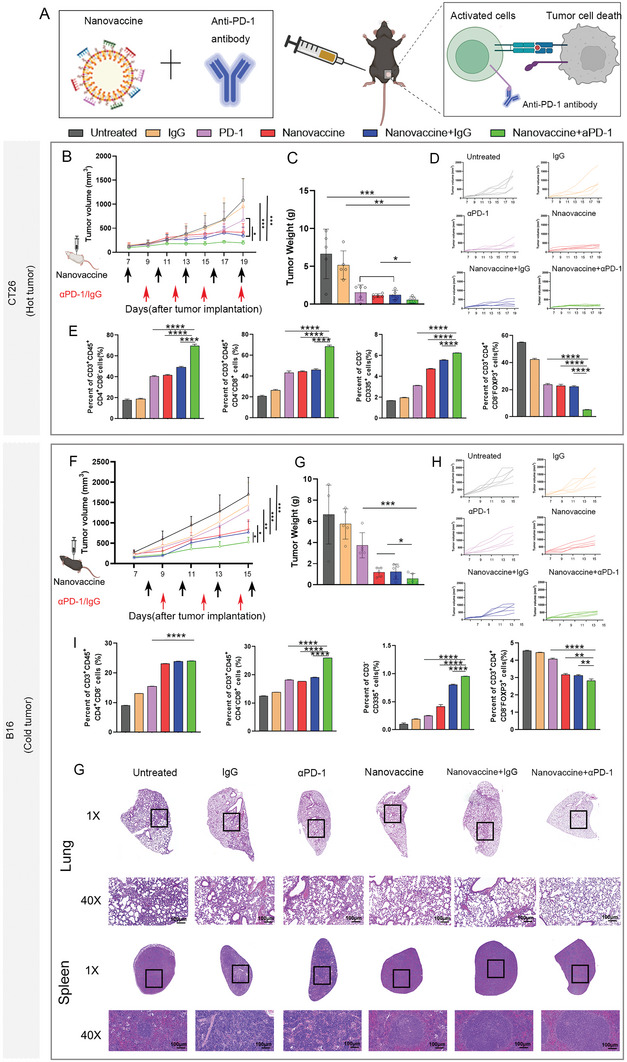
The nanovaccine enhanced the immune response to αPD‐1 therapy by reprogramming the immunosuppressive TME. A) Schematic illustration of the synergistic antitumor effects of the nanovaccine and αPD‐1 cancer immunotherapy. B‐E) Experiments in CT26 tumor‐bearing mice. B) Treatment schedule for the nanovaccine combined with αPD‐1 therapy, and average tumor growth curves of the tumors in groups receiving different treatments. C) Tumor weights in different treatment groups. D) Individual tumor growth curves in different treatment groups. E) Flow cytometry analysis of immune cell populations. F,G) Experiments in B16F10 tumor‐bearing mice. F) Treatment schedule for the nanovaccine combined with αPD‐1 therapy, and average tumor growth curves of the tumors receiving different treatments. G) Tumor weights in different treatment groups. H) Individual tumor growth curves in different treatment groups. I) Flow cytometry analysis of immune cell populations. G) H&E staining of lungs and spleens collected from mice after receiving different treatments.

The programmed death ligand‐1/programmed death‐1 (PD‐L1/PD‐1) signaling pathway is an important component of suppressive TME, which can limit the activation of T cells and enhance the immune escape to of tumor cells.^[^ ^61^ ^]^ Therefore, targeting the PD‐L1/PD‐1 pathway is an attractive strategy for cancer treatment; however, Treg accumulation and CD8^+^T depletion made the therapeutic effectiveness of PD‐L1/PD‐1 remains poor.^[^ ^62^, ^63^ ^]^ We were encouraged by the finding that our nanovaccine could relieve the immunosuppression in the TME and generate a more immunogenic phenotype. We hypothesized that our nanovaccine could improve anti‐PD‐1 (αPD‐1) efficacy modifying the TME T‐cell infiltration. Subsequently, αPD‐1 therapy was used to inhibit T‐cell exhaustion and further amplify the antitumor immune response (**Figure** **7**A). The therapeutic efficacy of the nanovaccine combined with αPD‐1 therapy was investigated in CT26 and B16F10 models. And IgG2a as the isotype control of PD‐1 antibody and selected as a negative control. Mice treated with the cocktail nanovaccine plus αPD‐1 therapy exhibited the most effective inhibition of tumors as well as the lightest tumor weight, suggesting that the nanovaccine and αPD‐1 cotreatment therapies had a synergistic effect (Figure 7B–D,F–H). Next, we detected the infiltration of immune cells in tumors. As assessed by flow cytometry, the proportions of CD4^+^ T, CD8^+^ T, and NK cells in mice treated with the cocktail nanovaccine+ αPD‐1 therapy were much higher (Figure 7E,I). Furthermore, the percentage of Treg cells was reduced in the TME after cocktail nanovaccine+ αPD‐1 treatment (Figure 7E,I). Noticeably, αPD‐1 therapy has a low response rate in melanoma patients.^[^ ^64^, ^65^ ^]^ We speculated that our combination nanovaccine would sensitize B16F10 tumors to ICB therapy, possibly by activating T cells and altering the TME in the B16F10 model into the infiltrated‐inflamed phenotype. Furthermore, we assessed the lungs, the primary metastatic site of the B16F10 model, to monitor the development of metastatic nodules. Hematoxylin and eosin staining revealed detectable metastatic cancer cells in the lung and spleen, while the cocktail nanovaccine and the cocktail nanovaccine+αPD‐1 combination treatment groups lacked metastasis. Interestingly, we observed a similar situation in the spleen. Inefficient T‐cell access to the TME is a cause of tumor resistance to ICB^[^ ^32^ ^]^; thus, regulating the function of TME effector cells may be a useful strategy to combine with ICB therapy. Taken together, these data indicate that the cocktail nanovaccine plus αPD‐1 treatment is an effective novel combination strategy.

## 3 Conclusion

In summary, as a proof of concept, we constructed an in situ nanovaccine system employing DP7‐C that not only facilitates siRNA cocktail delivery but also boosts antitumor immunity for cancer immunotherapy. We showed that the cocktail nanovaccine induces tumor cell apoptosis to release TAAs and enhances the maturation of DCs, which causes strong cross‐presentation of tumor neoantigens and more robust stimulation of effector T cells. Thus, our results demonstrate that the cocktail nanovaccine improved the immune context of the TME, successfully promoting antitumor immunity and converting the cold TME into a hot TME. Additionally, combination of the nanovaccine with αPD‐1 therapy induced significant inhibition of tumor growth. Our rationally designed nanovaccine can easily be adapted to encapsulate tumor factors or used for combined administration with other ICB agents or therapeutics. We predict that the characteristics of nanovaccines will allow them to be loaded with multiple targets, facilitating their use in the treatment of many complex diseases.

## 4 Experimental Section

### Material Synthesis and Characterization

DP7 was synthesized by the standard Fmoc solid‐phase peptide synthesis method on an automatic peptide synthesizer (CSBio 136XT); DP7‐C was generated using hydrophobic modification and purification of DP7 with cholesterol. siRNA was synthesized by GenePharma (Shanghai, China), and the sequences were listed in Table S1 (Supporting Information). The particle size and zeta potential of the nanoparticles were measured using a Malvern laser particle size analyzer (Nano‐Zs 90, Malvern). The morphology of nanoparticles was observed with transmission electron microscopy (TEM).

### Cell Culture

CT26, B16F10, and 293T cells were purchased from the American Type Culture Collection (ATCC). In brief, CT26 cells were cultured in RPMI‐1640 medium, and 293T and B16F10 cells were grown in DMEM. All media were supplemented with 10% fetal bovine serum (FBS), 100 units mL^−1^ penicillin, and 100 units mL^−1^ streptomycin at 37 °C in 5% CO_2. Bone marrow‐derived DCs (BMDCs) were obtained from 6‐week‐old C57BL/6J female mice according to our previous protocol.^[^ ^34^ ^]^ In brief, red blood cell lysis buffer was used to remove red blood cells, and RPMI‐1640 medium containing 10% FBS and 20 ng mL^−1^ granulocyte‐macrophage colony‐stimulating factor (GM‐CSF) were added to 3 × 10^6^ mouse bone marrow cells. After 8 days of culture, the BMDCs were collected for use.

### Agarose Gel Electrophoresis Assay

An agarose gel retardation assay was performed to detect the ability of DP7‐C to bind to small nucleic acids. After incubation of gradient proportions of DP7‐C with 0.5 µg of siRNA or CpG ODNs for 10 min at room temperature, electrophoresis was performed at 110 V for another 10 min. For the RNase protection assay, 0.5 µg of naked siRNA or DP7‐C/siRNA was mixed with 0.5 µl of RNase A (1 mg mL^−1^, ST578, Beyotime) and incubated for 0 to 4 h. For determination of long‐term stability, DP7‐C/siRNA (0.5 µg siRNA) was stored at 4 °C or room temperature for 1 to 4 weeks. After mixing the siRNA with 0.5% SDS for 10 min, a retardation experiment was conducted to assess the long‐term stability.

### In Vitro siRNA Transfection

The siRNA transfection efficiency was carried out in B16F10 cells. Scramble siRNA was labeled with FITC, Cy3 and Cy5. Cancer cells were seeded in 12‐well plates at a density of 1.0 × 10^4^ cells per well and then cultured at 37 °C for 12 h. The culture medium in the plates was replaced with new DMEM before transfection, and DP7‐C/siRNA at various combination were prepared. Thereinto, three groups were designed to demonstrate DP7‐C as a deliver system loading multiple small nucleic acids:^[^ ^1^ ^]^ single group (DP7‐C/FITC‐siRNA, containing 0.9 µg FITC‐siRNA);^[^ ^2^ ^]^ double group (DP7‐C/FITC‐siRNA+Cy3‐siRNA, containing 0.45 µg FITC‐siRNA and 0.45 µg Cy3‐siRNA);^[^ ^3^ ^]^ triple group (DP7‐C/FITC‐siRNA+Cy3‐siRNA+Cy5‐siRNA, containing 0.3 µg FITC‐siRNA, 0.3 µg Cy3‐siRNA and 0.3 µg Cy5‐siRNA). The different siRNA was mixed at DMEM initially, and then, 4.5 µg DP7‐C was added in the siRNA mixture and incubated for 15 min at room temperature in dark. Next, the culture medium was replaced with fresh complete DMEM and then incubated for another 24 h. The luciferase level in each well was measured according to the manufacturer's instructions.

### In Vitro Cellular Uptake and Intracellular Localization of the Nanovaccine

CT26 and B16F10 cells (1 × 10^4^ cells per well) were plated on cell slides and then transfected with FAM‐NC (FAM‐labeled scramble siRNA) in the medium. After the indicated incubation times (6 and 24 h), the cells were washed with 1x PBS and then fixed with 4% paraformaldehyde. Then, the samples were permeabilized for 10 min with 0.3% Triton X‐100 and blocked with 5% BSA solution. After 30 min of blocking, the samples were incubated with the following antibodies: EE1A (1:100, Invitrogen) and LAMP‐1‐PE (1:100, Invitrogen). LysoTracker Red (Beyotime Biotechnology) was used to detect lysosomes. After FAM‐NC was transfected for 4 and 24 h, cell slides were incubated with LysoTracker Red for 2 h at room temperature. All slides were counterstained with 4,6‐diamidino‐2‐phenylindole (DAPI, D9542‐1MG, Sigma‐Aldrich) for 3–5 min, and the colocalization degree was determined utilizing a confocal laser scanning microscope (Olympus 1000, Japan).

### RNA Encapsulation Efficiency Detection

The RNA encapsulation efficiency was determined by the Quant‐iT RiboGreen Assay (R11491, Invitrogen). Quantification of RNA in DP7‐C was conducted using a standard curve generated from a dilution series of the corresponding RNA stock. Fluorescence was measured using a fluorescence microplate reader (BioTek) set at 480‐nm excitation and 520‐nm emission.

### In Vitro Gene Silencing

Cells were seeded into 6‐well plates at 1 × 10^5^ cells well^−1^ and cultured for 24 h. Then cells were transfected with DP7‐C, DP7‐C/si‐Scramble (containing 3 µg si‐Scramble), DP7‐C/si‐Stat3 (containing 3 µg si‐Stat3), DP7‐C/si‐Ccr2 (containing 3 µg si‐Ccr2), DP7‐C/si‐Tgfβ (containing 3 µg si‐ Tgfβ), DP7‐C/si‐Stat3+ si‐Ccr2+ si‐Tgfβ (containing 1 µg si‐Stat3, 1 µg si‐Ccr2 and 1 µg si‐Tgfβ) in DMEM for 6 h. Then the medium was added 1 mL complete medium and cultured for additional 42 h. After that, the expression levels of STAT3, CCR2 and TGFβ were analyzed by qPCR and western blotting. Detailed information regarding primers for qPCR was shown in Table S2 (Supporting Information).

### Western Blotting

Tumor cells and tissues were collected and lysed with RIPA buffer (P0013B, Beyotime) added with protease inhibitor cocktail (P5726, Sigma–624 Aldrich). Equal amounts of protein measured by the BCA Protein Assay Kit (P0010, Beyotime) were mixed with loading buffer (P0015, Beyotime) and boiled at 100 °C for 10 min. Next, the proteins were denatured at 95 °C for 10 min. The denatured proteins were separated into 10% SDS‐polyacrylamide gels and then transferred to polyvinylidene difluoride (PVDF) membranes (Millipore, USA). The membranes were blocked with 5% skimmed milk for 1 h at room temperature and incubated in primary antibody solution at 4 °C overnight. Subsequently, the membranes were washed with 1 × TBST three times. Then, the members were incubated with secondary antibody. Finally, the immunoblots were developed using Thermo Scientific™ Pierce™ ECL Western Blotting Substrate (Thermo Fisher, MA, USA) and photographic film. Detailed information on the primary antibodies and secondary antibodies used for Western blotting was listed in Table S3 (Supporting Information).

### Cell Viability Assay

To evaluate the cytotoxicity of DP7‐C, 293T cells were extracted and seeded in a 96‐well plate at a density of 1*10^4^ cells per well and incubated for 24 h. Cationic liposomes were added in the concentration range of 0–200 µg mL^−1^. After incubation for 24 h, the cells were washed with 1 × PBS, and then fresh medium containing 10% Cell Counting Kit‐8 (CCK‐8; 96992, Sigma, UK) solution was added to each well. After incubation for 40 min, the absorption at 450 nm was measured using a microplate reader.

### Evaluation of Systemic Cytokine Levels

Mouse blood was collected via retro‐orbital bleeding and placed in a 37 °C incubator for 1 h. Then, the blood was centrifuged at 13 000 rpm for 15 min, and collected the supernatant. The samples were stored at −20 °C for ELISA detection. All samples were diluted in a gradient and measured over time by using ELISA kits for IL‐12p70 (ab208348, Abcam), IL‐6 (ab100713, Abcam), IFN‐γ (ab213866, Abcam), and TNF‐α (EK0527, Boster) according to a standard protocol. All samples were measured in triplicate.

### Tissue Section Staining Analysis

Tumor tissues and other organs were fixed with 4% paraformaldehyde, embedded in paraffin, and cut into 5 µm sections. The tissue sections were subjected to antigen retrieval and serum blocked for 30 min. The tissues were incubated with primary antibodies in the Table S3 (Supporting Information), and the signals were detected by immunohistochemistry or immunofluorescence.

For multiplexed IHC (mIHC), tissue sections were also subjected to antigen retrieval and serum blocked for 30 min. Primary antibody was added, and the sections were incubated at RT for 60 min (Table S3, Supporting Information). Then, the antibody solution was removed, and the sections were washed with 1× TBST two times for 3 min each time. Next. Sections were covered with SignalStain Boost IHC detection Reagent (CST, HRP rabbit, #8114 or HRP mouse, #8125) specific to the species of the primary antibody and incubated at RT for 30 min in the dark. The slides were washed with gentle agitation in the dark two times. The fluorophore‐conjugated TSA Plus amplification reagent was diluted per the manufacturer's recommendation and was applied to each slide and incubated for 10 min at RT in the dark. The slides were washed with two times with gentle agitation in the dark. Subsequently, the slides were boiled in 1 mM EDTA, pH 8.0, and then maintained at a subboiling temperature for 10 min. After the slides were cooled down, steps were repeated to stain with the next antibody. The slides were incubated with Opal DAPI for 10 min and washed with three times. The PerkinElmer Vectra3 platform was used to scan and image slides. TLSs were selected and batch analyzed using PerkinElmer Inform software.

2.12 Generation of and testing of the cocktail vaccine in CT26 and B16F10 model mice: CT26 cells (1 × 10^6^) and B16F10 cells (5 × 10^5^) were subcutaneously injected into the flanks of Balb/c or C57BL/6J mice. One week later, the mice were randomly divided into 13 groups (*n* = 5). The 13 groups were injected with saline, DP7‐C/si‐Scramble (containing 12 µg si‐Scramble), DP7‐C/si‐Stat3 (containing 12 µg si‐Stat3), DP7‐C/si‐Ccr2 (containing 12 µg si‐Ccr2), DP7‐C/si‐Tgfβ (containing 12 µg si‐Tgfβ), DP7‐C/CpG ODNs (containing 12 µg CpG ODNs), DP7‐C/si‐Stat3/CpG ODNs (containing 6 µg si‐Stat3 and 6 µg CpG ODNs), DP7‐C/si‐Stat3+si‐Ccr2 (containing 6 µg si‐Stat3 and 6 µg si‐Ccr2), DP7‐C/si‐Stat3+si‐Tgfβ (containing 6 µg si‐Stat3 and 6 µg si‐Tgfβ), DP7‐C/si‐Ccr2/CpG ODNs (containing 6 µg si‐Ccr2 and 6 µg CpG ODNs), DP7‐C/si‐Ccr2+si‐Tgfβ(containing 6 µg si‐Ccr2 and 6 µg si‐Tgfβ), DP7‐C/si‐Tgfβ/CpG ODNs (containing 6 µg si‐Tgfβ and 6 µg CpG ODNs), or DP7‐C/si‐Stat3+si‐Ccr2+ si‐Tgfβ/CpG ODNs (Nanovaccine, containing 3 µg si‐Stat3, 3 µg si‐Ccr2, 3 µg si‐Tgfβ and 3 µg CpG ODNs). The tumor volume was recorded and calculated with the formula tumor volume = length × width × width/2. At the end of the experiment, all mice were decapitated. The tumors and organs of the mice were collected.

For the groups receiving the nanovaccine (containing 3 µg si‐Stat3, 3 µg si‐Ccr2, 3 µg si‐Tgfβ and 3 µg CpG ODNs) combined with an immune checkpoint inhibitor, αPD‐1 (200 µg per mouse) was administered intraperitoneally the day after intertumoral administration of the nanovaccine. The detailed information was presented in Figure 7B,F.

### Transcriptome Sequencing

The tumor samples collected from mice were washed in 1X PBS. Total RNA was extracted from tumor samples using the Dynabeads mRNA Purification Kit (61006, Thermo Fisher Scientific), and RNA quality was evaluated using the Agilent 2100 Bioanalyzer system (Agilent Technologies). An mRNA sequencing library was constructed, and the Illumina HiSeq 4000 Sequencing System (Illumina) was used for paired‐end sequencing. Signaling pathway annotation analysis of differentially expressed genes (DEGs) was performed with Kyoto Encyclopedia of Genes and Genomes (KEGG, https://www.genome.jp/kegg/).

### Postoperative Models and Treatment

To investigate the ability of the nanovaccine to prevent recurrence, 1 × 10^6^ B16F10 cells were transplanted into C57BL/6J mice. After seven days, the mice were randomly divided into two groups (*n* = 5), and tumors were resected when the tumor size reached 100 cm^3^. To mimic residual micro tumors after surgery, ≈1% of the tumor volume was retained. After three days of rest, the two groups were treated with saline or the nanovaccine at the surgical site. The detailed administration information was shown in Figure 6A. The tumor size parameters were measured every two days, and tumor size was calculated with a specific formula.

### Flow Cytometry Analysis

Single‐cell suspensions were obtained from cultured cells or tumor tissues. The tumors were enzymatically digested in 1 mg mL^−1^ collagenase I and 1 mg mL^−1^ collagenase IV. Fixable Viability Stain 700 (564997, BD) was used to exclude dead cells. The following antibodies were used for surface staining: CD3e‐APC‐Cy™7 (557596, BD), CD8a‐PerCP‐Cy™5.5 (551162, BD), CD335‐BV421 (562850, BD), CD11b‐FITC (557396, BD), CD80‐APC (ab95549, Abcam), CD86‐PerCP‐Cy5.5 (105027, Biolegend), Gr‐1‐APC (108411, Biolegend) and CD11c‐FITC (117306, Biolegend). After being stained at room temperature in the dark, the cells were fixed with 4% methanol, permeabilized with 0.5% Triton, and intracellularly stained with Foxp3‐Alexa Fluor 647 (560401, BD). A BD FACSymphony A5 instrument was utilized for flow cytometry, and NovoExpress Software (Tree Star) was employed to analyze the data. Cell populations were identified as follows: CD4^+^ T cells: live/CD45^+^/CD3^+^/CD4^+^/FoxP3^−^; CD8^+^ T cells: live/CD45^+^/CD3^+^/CD8^+^; Treg cells: live/CD45^+^/CD3^+^/CD4^+^/FoxP3^+^; natural killer (NK) cells: live/CD45^+^/CD3^−^/NKP46^+^; MDSC cells: live/ CD11b^+^/Gr1^+^ and DCs: live/CD45^+^/CD3^−^/CD11c^+^.

### Bilateral Murine Tumor Model

Eight‐week‐old female Balb/c and C57BL/6J mice received subcutaneous injection of CT26 cells in the right (local) and left (distant) flanks. At 7 days postimplantation, the nanovaccine (containing 3 µg si‐Stat3, 3 µg si‐Ccr2, 3 µg si‐Tgfβ and 3 µg CpG ODNs) was intratumorally injected into only the right tumor, while the left (distant) tumor was left untreated. The nanovaccine was administered every 3 days. Both the local and distant tumors in mice were measured every 2 days, and tumor volume was calculated with a specific formula. Survival was also monitored.

### Ethical Approval

The animal experiments and human subjects were approved by the Experimental Animal Management and Ethics Committee of West China Hospital, Sichuan University. All animal procedures complied with the Animal Care and Use Committee of Sichuan University.

### Statistical Analysis

All animal experiments were randomized, and the data were analyzed using unpaired two‐tailed Student's t test by Prism 8 (GraphPad Software, Inc., La Jolla, CA, USA). *p*<0.05 was considered statistically significant.

## Conflict of Interest

The authors declare no conflict of interest.

## Supporting information

Supporting Information

## Data Availability

The data that support the findings of this study are available from the corresponding author upon reasonable request.

## References

[1] A. Dhiman , R. Sharma , R. K. Singh , Acta Pharm. Sin. B 2022, 12, 3006. 35865090 10.1016/j.apsb.2022.03.021 PMC9293743

[2] A. Cercek , C. S. D. Roxburgh , P. Strombom , J. J. Smith , L. K. F. Temple , G. M. Nash , J. G. Guillem , P. B. Paty , R. Yaeger , Z. K. Stadler , K. Seier , M. Gonen , N. H. Segal , D. L. Reidy , A. Varghese , J. Shia , E. Vakiani , A. J. Wu , C. H. Crane , M. J. Gollub , J. Garcia‐Aguilar , L B. Saltz , M. R. Weiser , JAMA Oncol 2018, 4, e180071. 29566109 10.1001/jamaoncol.2018.0071 PMC5885165

[3] A. M. Tsimberidou , E. Fountzilas , M. Nikanjam , R. Kurzrock , Cancer Treat. Rev. 2020, 86, 102019. 32251926 10.1016/j.ctrv.2020.102019 PMC7272286

[4] Q. He , X. Jiang , X. Zhou , J. Weng , J. Hematol. Oncol. 2019, 12, 139. 31852498 10.1186/s13045-019-0812-8 PMC6921533

[5] L. Li , S. P. Goedegebuure , W. E. Gillanders , Ann. Oncol. 2017, 28, xii11. 29253113 10.1093/annonc/mdx681 PMC5834106

[6] K. G. K. Deepak , R. Vempati , G. P. Nagaraju , V. R. Dasari , N. S , D. N. Rao , R. R. Malla , Pharmacol Res 2020, 153, 104683. 32050092 10.1016/j.phrs.2020.104683

[7] C. Hutchings , J. A. Phillips , M. B. A. Djamgoz , Biochim. Biophys. Acta, Rev. Cancer 2020, 1874, 188411. 32828885 10.1016/j.bbcan.2020.188411

[8] T. Wu , Y. Dai , Cancer Lett 2017, 387, 61. 26845449 10.1016/j.canlet.2016.01.043

[9] A. Ribas , J D. Wolchok , Science 2018, 359, 1350. 29567705 10.1126/science.aar4060 PMC7391259

[10] Q. Wu , L. Jiang , S. C. Li , Q. J. He , B. Yang , J. Cao , Acta Pharmacol. Sin. 2021, 42, 1. 32152439 10.1038/s41401-020-0366-x PMC7921448

[11] E. Ylösmäki , V. Cerullo , Curr. Opin. Biotechnol. 2020, 65, 25. 31874424 10.1016/j.copbio.2019.11.016

[12] O. Hemminki , J. M. Dos Santos , A. Hemminki , J. Hematol. Oncol. 2020, 13, 84. 32600470 10.1186/s13045-020-00922-1 PMC7325106

[13] J. Tang , L. Pearce , J. O'Donnell‐Tormey , V M. Hubbard‐Lucey , Nat Rev Drug Discov 2018, 17, 783. 30337722 10.1038/nrd.2018.167

[14] L. Galluzzi , J. Humeau , A. Buqué , L. Zitvogel , G. Kroemer , Nat. Rev. Clin. Oncol. 2020, 17, 725. 32760014 10.1038/s41571-020-0413-z

[15] H R. Cha , J H. Lee , S. Ponnazhagan , Cancer Res. 2020, 80, 1615. 32066566 10.1158/0008-5472.CAN-19-2948 PMC7641094

[16] D. A. Braun , Z. Bakouny , L. Hirsch , R. Flippot , E. M. Van Allen , C. J. Wu , T. K. Choueiri , Nat. Rev. Clin. Oncol. 2021, 18, 199. 33437048 10.1038/s41571-020-00455-z PMC8317018

[17] R. Kuai , X. Sun , W. Yuan , L. J. Ochyl , Y. Xu , A. Hassani Najafabadi , L. Scheetz , M. Z. Yu , I. Balwani , A. Schwendeman , J. J. Moon , J. Control Release 2018, 282, 131. 29702142 10.1016/j.jconrel.2018.04.041 PMC6056764

[18] L. Bialkowski , K. Van der Jeught , S. Bevers , P. Tjok Joe , D. Renmans , C. Heirman , J. L. Aerts , K. Thielemans , Int. J. Cancer 2018, 143, 686. 29464699 10.1002/ijc.31331

[19] Y. Wang , L. Zhang , Z. Xu , L. Miao , L. Huang , Mol. Ther. 2018, 26, 420. 29249397 10.1016/j.ymthe.2017.11.009 PMC5835019

[20] L A. Phylactou , Adv Drug Deliv Rev 2000, 44, 97. 11072108 10.1016/s0169-409x(00)00088-0

[21] Y. Yuan , Z. Gu , C. Yao , D. Luo , D. Yang , Small 2019, 15, 1900172. 10.1002/smll.201900172 30972963

[22] C M. Jewell , D M. Lynn , Adv Drug Deliv Rev 2008, 60, 979. 18395291 10.1016/j.addr.2008.02.010 PMC2476211

[23] D. S. Spencer , A. B. Shodeinde , D. W. Beckman , B. C. Luu , H. R. Hodges , N. A. Peppas , J Control Release 2021, 332, 608. 33675879 10.1016/j.jconrel.2021.03.004 PMC8089052

[24] J. A. Kulkarni , D. Witzigmann , S. Chen , P. R. Cullis , R. van der Meel , Acc. Chem. Res. 2019, 52, 2435. 31397996 10.1021/acs.accounts.9b00368

[25] S. Yonezawa , H. Koide , T. Asai , Adv Drug Deliv Rev 2020, 154, 64. 32768564 10.1016/j.addr.2020.07.022 PMC7406478

[26] Y. Xiao , D. Yu , Pharmacol. Ther. 2021, 221, 107753. 33259885 10.1016/j.pharmthera.2020.107753 PMC8084948

[27] D. Pissuwan , T. Niidome , M B. Cortie , J Control Release 2011, 149, 65. 20004222 10.1016/j.jconrel.2009.12.006

[28] T F. Gajewski , H. Schreiber , Y X. Fu , Nat. Immunol. 2013, 4, 1014. 10.1038/ni.2703 PMC4118725 24048123

[29] M. Binnewies , E. W. Roberts , K. Kersten , V. Chan , D. F. Fearon , M. Merad , L. M. Coussens , D. I. Gabrilovich , S. Ostrand‐Rosenberg , C. C. Hedrick , R. H. Vonderheide , M. J. Pittet , R. K. Jain , W. Zou , T. K. Howcroft , E. C. Woodhouse , R. A. Weinberg , M. F. Krummel , Nat. Med. 2018, 24, 541. 29686425 10.1038/s41591-018-0014-x PMC5998822

[30] J. Kim , J. Hong , J. Lee , S. Fakhraei Lahiji , Y. H. Kim , J. Control Release 2021, 332, 109. 33571549 10.1016/j.jconrel.2021.02.002

[31] P. A. Zucali , C. C. Lin , B. C. Carthon , T. M. Bauer , M. Tucci , A. Italiano , R. Iacovelli , W. C. Su , C. Massard , M. Saleh , G. Daniele , A. Greystoke , M. Gutierrez , S. Pant , Y. C. Shen , M. Perrino , R. Meng , G. Abbadessa , H. Lee , Y. Dong , M. Chiron , R. Wang , L. Loumagne , L. Lépine , J. de Bono , J. Immunother. Cancer 2022, 10, e003697. 35058326 10.1136/jitc-2021-003697 PMC8783811

[32] W. J. Ho , E. M. Jaffee , L. Zheng , Nat. Rev. Clin. Oncol. 2020, 17, 527. 32398706 10.1038/s41571-020-0363-5 PMC7442729

[33] H. Fang , Y A. Declerck , Cancer Res. 2013, 73, 4965. 23913938 10.1158/0008-5472.CAN-13-0661 PMC3815577

[34] B. Zhao , B. Zhou , K. Shi , R. Zhang , C. Dong , D. Xie , L. Tang , Y. Tian , Z. Qian , L. Yang , Cancer Sci. 2021, 112, 2481. 33792132 10.1111/cas.14903 PMC8177784

[35] R. Zhang , L. Tang , Y. Tian , X. Ji , Q. Hu , B. Zhou , D. Zhenyu , X. Heng , L. Yang , Biomaterials 2020, 241, 119852. 32120313 10.1016/j.biomaterials.2020.119852

[36] S. Spranger , H. K. Koblish , B. Horton , P. A. Scherle , R. Newton , T. F. Gajewski , J Immunother Cancer 2014, 2, 3. 24829760 10.1186/2051-1426-2-3 PMC4019906

[37] M F. Sanmamed , L. Chen , Cell 2019, 176, 677. 30682374 10.1016/j.cell.2019.01.008

[38] D S. Chen , I. Mellman , Nature 2017, 541, 321. 28102259 10.1038/nature21349

[39] J A. Joyce , D T. Fearon , Science 2015, 348, 74. 25838376 10.1126/science.aaa6204

[40] A. Z. Siddiquee , K. Turkson , Cell Res. 2008, 18, 254. 18227858 10.1038/cr.2008.18 PMC2610254

[41] M. R. Fein , X. Y. He , A. S. Almeida , E. Bružas , A. Pommier , R. Yan , A. Eberhardt , D. T. Fearon , L. Van Aelst , J. E. Wilkinson , C. O. Dos Santos , M. Egeblad , J. Exp. Med. 2020, 217, e20181551. 32667673 10.1084/jem.20181551 PMC7537399

[42] E. Batlle , J. Massagué , Immunity 2019, 50, 924. 30995507 10.1016/j.immuni.2019.03.024 PMC7507121

[43] H. Yu , H. Lee , A. Herrmann , R. Buettner , R. Jove , Nat. Rev. Cancer 2014, 14, 736. 25342631 10.1038/nrc3818

[44] H. Yu , M. Kortylewski , D. Pardoll , Nat. Rev. Immunol. 2007, 7, 41. 17186030 10.1038/nri1995

[45] N. S. Nagathihalli , J. A. Castellanos , C. Shi , Y. Beesetty , M. L. Reyzer , R. Caprioli , X. Chen , A. J. Walsh , M. C. Skala , H. L. Moses , N. B. Merchant , Gastroenterology 2015, 149, 1932. 26255562 10.1053/j.gastro.2015.07.058 PMC4863449

[46] M. Xu , Y. Wang , R. Xia , Y. Wei , X. Wei , Cell Prolif 2021, 54, e13115. 34464477 10.1111/cpr.13115 PMC8488570

[47] X. Li , W. Yao , Y. Yuan , P. Chen , B. Li , J. Li , R. Chu , H. Song , D. Xie , X. Jiang , H. Wang , Gut 2017, 66, 157. 26452628 10.1136/gutjnl-2015-310514

[48] S. Li , M. Liu , M. H. Do , C. Chou , E. G. Stamatiades , B. G. Nixon , W. Shi , X. Zhang , P. Li , S. Gao , K. J. Capistrano , H. Xu , N. V. Cheung , M. O. Li , Nature 2020, 587, 121. 33087933 10.1038/s41586-020-2850-3 PMC8353603

[49] M. Gestin , M. Dowaidar , Ü. Langel , Adv. Exp. Med. Biol. 2017, 1030, 255. 29081057 10.1007/978-3-319-66095-0_11

[50] L. Galluzzi , A. Buqué , O. Kepp , L. Zitvogel , G. Kroemer , Nat. Immunol. 2022, 23, 487. 35145297 10.1038/s41590-022-01132-2

[51] Y. Li , H. Zhang , Q. Li , P. Zou , X. Huang , C. Wu , L. Tan , Cancer Lett 2020, 495, 12. 32941949 10.1016/j.canlet.2020.09.011

[52] W. Li , J. Yang , L. Luo , M. Jiang , B. Qin , H. Yin , C. Zhu , X. Yuan , J. Zhang , Z. Luo , Y. Du , Q. Li , Y. Lou , Y. Qiu , J. You , Nat. Commun. 2019, 10, 3349. 31350406 10.1038/s41467-019-11269-8 PMC6659660

[53] P. A. Roche , K. Furuta , Nat. Rev. Immunol. 2015, 15, 203. 25720354 10.1038/nri3818 PMC6314495

[54] D. V. Krysko , A. D. Garg , A. Kaczmarek , O. Krysko , P. Agostinis , P. Vandenabeele , Nat. Rev. Cancer 2012, 12, 860. 23151605 10.1038/nrc3380

[55] P. G. Coulie , B. J. Van den Eynde , P. van der Bruggen , T. Boon , Nat. Rev. Cancer 2014, 14, 135. 24457417 10.1038/nrc3670

[56] A. Cirella , C. Luri‐Rey , C. A. Di Trani , A. Teijeira , I. Olivera , E. Bolaños , E. Castañón , B. Palencia , D. Brocco , M. Fernández‐Sendin , F. Aranda , P. Berraondo , I. Melero , Pharmacol. Ther. 2022, 239, 108189. 35430292 10.1016/j.pharmthera.2022.108189

[57] M. A. Kursunel , G. Esendagli , Cytokine Growth Factor Rev. 2016, 31, 73. 27502919 10.1016/j.cytogfr.2016.07.005

[58] R. Siersbæk , V. Scabia , S. Nagarajan , I. Chernukhin , E. K. Papachristou , R. Broome , S. J. Johnston , S. E. P. Joosten , A. R. Green , S. Kumar , J. Jones , S. Omarjee , R. Alvarez‐Fernandez , S. Glont , S. J. Aitken , K. Kishore , D. Cheeseman , E. A. Rakha , C. D'Santos , W. Zwart , A. Russell , C. Brisken , J. S. Carroll , Cancer Cell 2020, 38, 412. 32679107 10.1016/j.ccell.2020.06.007 PMC7116707

[59] R. Weber , Z. Riester , L. Hüser , C. Sticht , A. Siebenmorgen , C. Groth , X. Hu , P. Altevogt , J. S. Utikal , V. Umansky , J Immunother Cancer 2020, 8, e000949. 32788238 10.1136/jitc-2020-000949 PMC7422659

[60] A. C. Huang , R. Zappasodi , Nat. Immunol. 2022, 23, 660. 35241833 10.1038/s41590-022-01141-1 PMC9106900

[61] X. Jiang , J. Wang , X. Deng , F. Xiong , J. Ge , B. Xiang , X. Wu , J. Ma , M. Zhou , X. Li , Y. Li , G. Li , W. Xiong , C. Guo , Z. Zeng , Mol Cancer 2019, 18, 10. 30646912 10.1186/s12943-018-0928-4 PMC6332843

[62] M. L. Dixon , L. Luo , S. Ghosh , J. M. Grimes , J. D. Leavenworth , J. W. Leavenworth , Mol Cancer 2021, 20, 150. 34798898 10.1186/s12943-021-01450-3 PMC8605582

[63] C. D'Alterio , M. Buoncervello , C. Ieranò , M. Napolitano , L. Portella , G. Rea , A. Barbieri , A. Luciano , G. Scognamiglio , F. Tatangelo , A. M. Anniciello , M. Monaco , E. Cavalcanti , P. Maiolino , G. Romagnoli , C. Arra , G. Botti , L. Gabriele , S. Scala , J Exp Clin Cancer Res 2019, 38, 432. 31661001 10.1186/s13046-019-1420-8 PMC6819555

[64] J. M. Zaretsky , A. Garcia‐Diaz , D. S. Shin , H. Escuin‐Ordinas , W. Hugo , S. Hu‐Lieskovan , D. Y. Torrejon , G. Abril‐Rodriguez , S. Sandoval , L. Barthly , J. Saco , B. Homet Moreno , R. Mezzadra , B. Chmielowski , K. Ruchalski , I. P. Shintaku , P. J. Sanchez , C. Puig‐Saus , G. Cherry , E. Seja , X. Kong , J. Pang , B. Berent‐Maoz , B. Comin‐Anduix , T G. Graeber , P C. Tumeh , T N. Schumacher , R S. Lo , A. Ribas , N. Engl. J. Med. 2016, 375, 819. 27433843 10.1056/NEJMoa1604958 PMC5007206

[65] A. Patnaik , W. J. Hwu , J. S. Weber , T. C. Gangadhar , P. Hersey , R. Dronca , R. W. Joseph , H. Zarour , B. Chmielowski , D. P. Lawrence , A. Algazi , N. A. Rizvi , B. Hoffner , C. Mateus , K. Gergich , J A. Lindia , M. Giannotti , X N. Li , S. Ebbinghaus , S P. Kang , C. Robert , JAMA, J. Am. Med. Assoc. 2016, 315, 1600.

