# Supplementary material for: In Situ Cocktail Nanovaccine for Cancer Immunotherapy
Source: Adv Sci (Weinh). 2023 Sep 22;10(31):2207697. doi: 10.1002/advs.202207697 (PMC10625102; doi:10.1002/advs.202207697)
Supplement: Supplementary file 1 — Supporting Information [file ADVS-10-2207697-s001.pdf]

ADVANCED SCIENCE
Open Access

Supporting Information

for *Adv. Sci.*, DOI 10.1002/advs.202207697

In Situ Cocktail Nanovaccine for Cancer Immunotherapy

*Mohan Liu, Daoyuan Xie, Die Hu, Rui Zhang, Yusi Wang, Lin Tang, Bailing Zhou, Binyan Zhao and Li Yang**

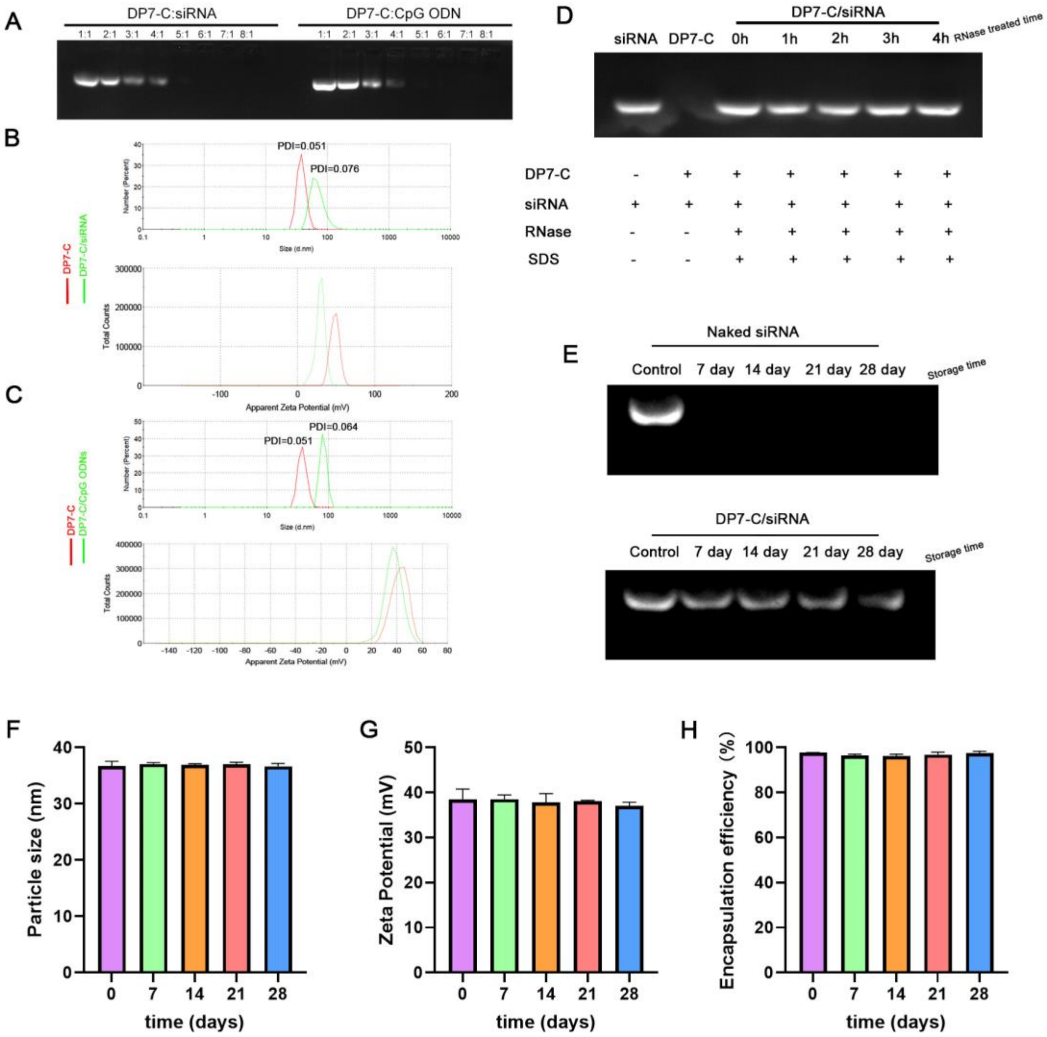

Figure S1. Characterization and stability of DP7-C carrying small nucleic acids. (A) Gel retardation analysis. (B and C) Particle size and zeta potential distributions of DP7- C, DP7-C/siRNA and DP7-C/CpG ODNs. (D) Stability of naked siRNA treated with RNaseA in vitro (mass ratio, 5:1). (E) Stability of small nucleic acids carried in DP7-C after incubation at room temperature for 1, 2, 3, and 4 weeks. F-H) Analytical testing DP7-C/siRNA stored at room temperature for 0-4 weeks. F) Particle size; G) zeta potential; H) Encapsulation efficiency.

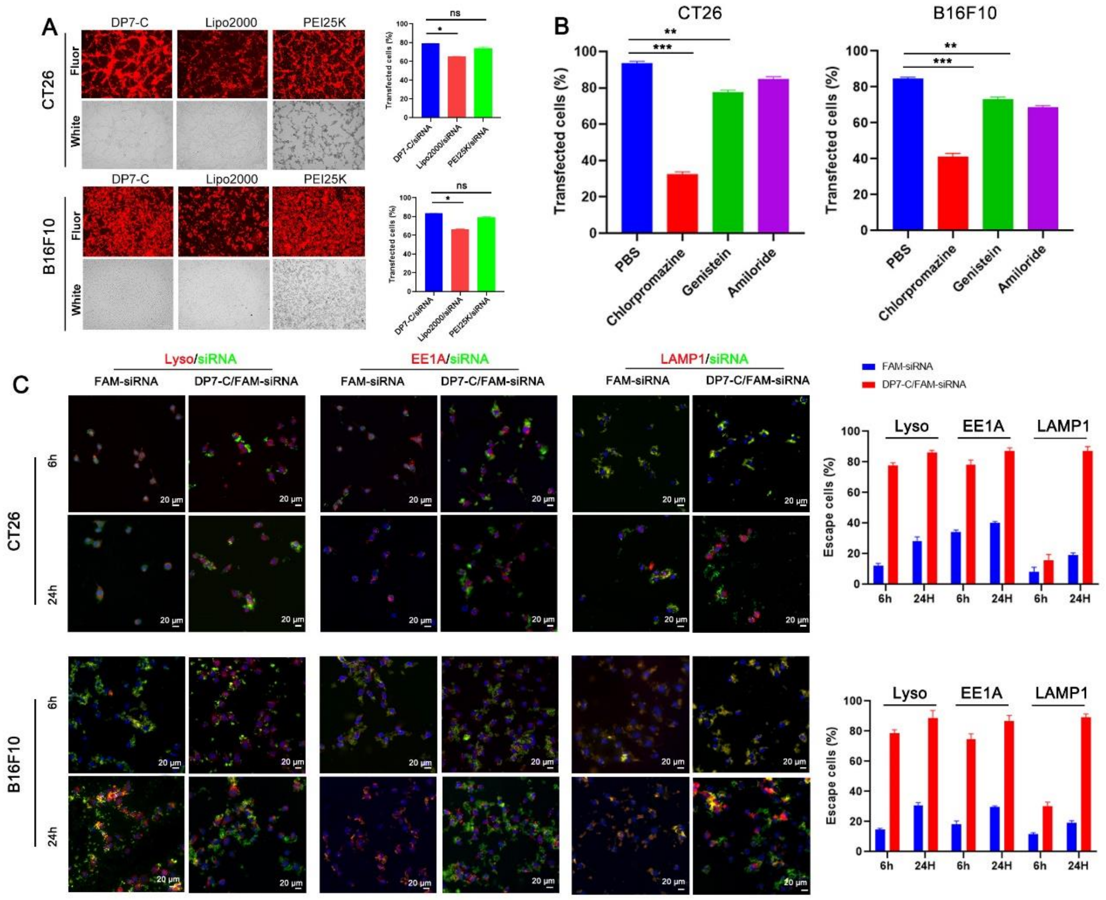

Figure S2. Intracellular delivery efficiency of DP7-C/siRNA. (A) Fluorescence images and flow cytometry analyze of CT26 and B16F10 cells transfected with Cy3-labeled siRNA via different vectors. (B) Transfection efficiency of DP7-C/Cy3-siRNA after treatment with three cell uptake inhibitors: amiloride (inhibitor of the macropinocytosis pathway), genistein (inhibitor of the caveolin-mediated pathway) and chlorpromazine (inhibitor of the clathrin-mediated pathway). (C) Cells were stained to detect lysosomes (red), EEA1 (red), and LAMP1 (red). Yellow indicates the colocalization of siRNA and endosomes.

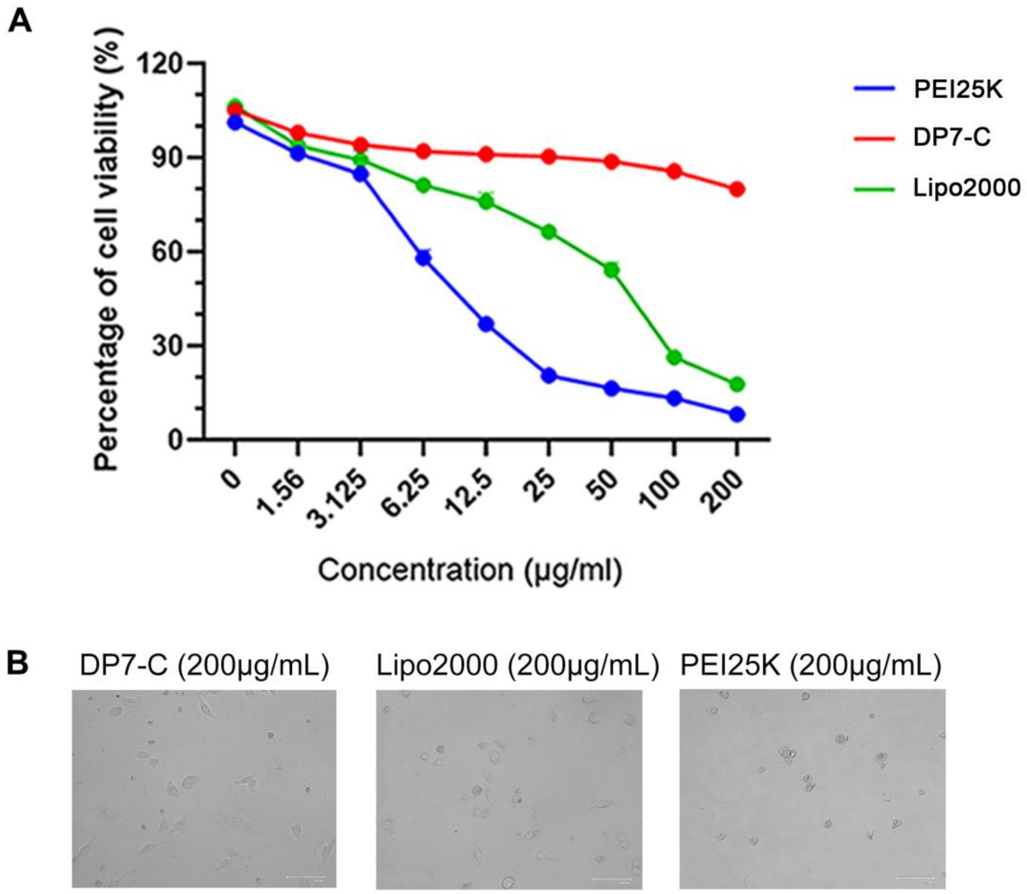

Figure S3. The cytotoxicity of DP7-C. (A) Relative cell viability after treatment with DP7-C, PEI25K, and Lipofectamine 2000. Data are expressed as the mean ± SD. (B) Morphological changes in the cells treated with various vectors.

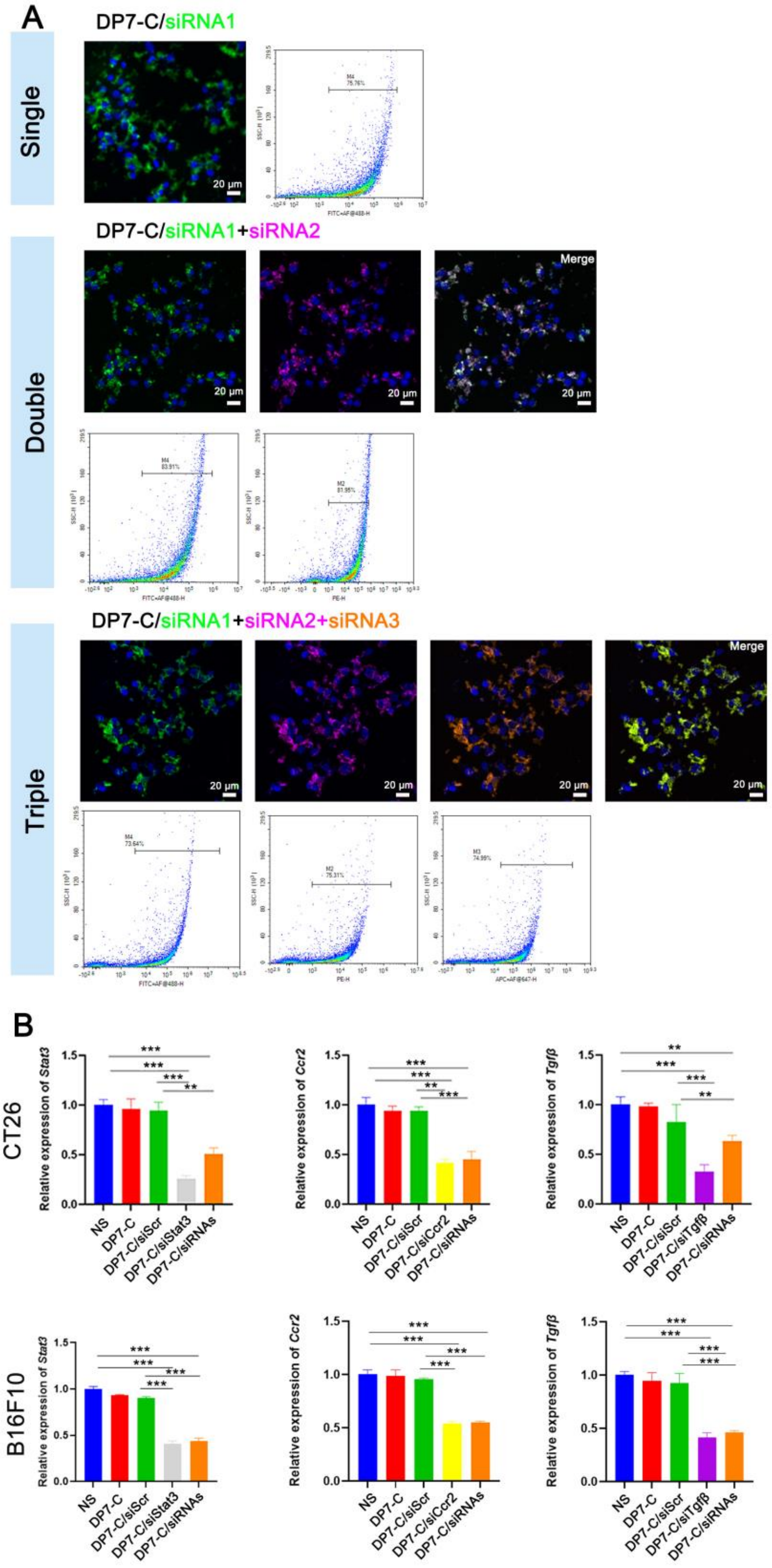

Figure S4. The transfected efficiency of DP7-C delivers multiple siRNAs. (A) Fluorescence images and flow cytometry analyze of transfected with single

(FITC-siRNA) or double (FITC-siRNA+Cy3-siRNA) or triple (FITC-siRNA+Cy3-siRNA+Cy5-siRNA) siRNA by DP7-C. (B) DP7-C delivery of target siRNAs transfected into tumor cells for 48 hours.

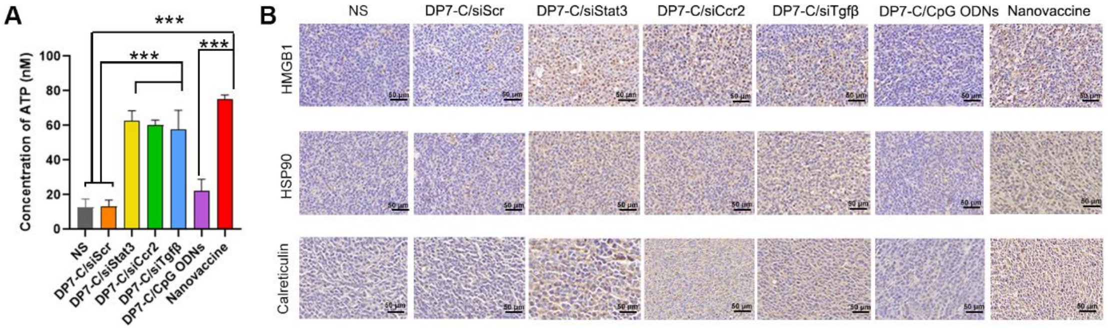

Figure S5. The immunogenic cell death induction upon nanovaccine treatment in B16F10 model. (A) Quantification of ATP levels in nanovaccine-treated B16F10 cells. (B) In vivo immunogenic cell death induction upon nanovaccine treatment in B16F10-bearing mice.

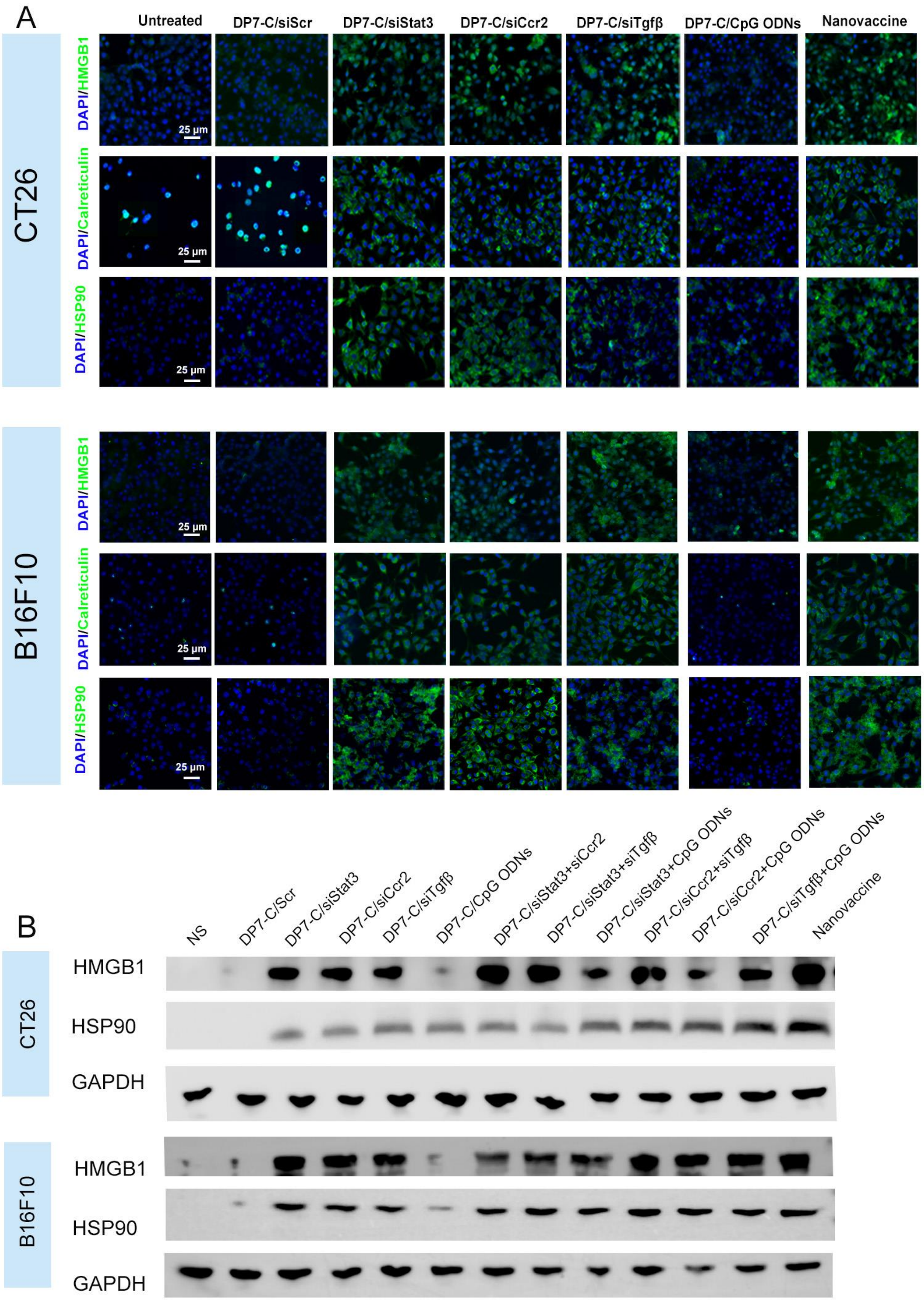

Figure S6. Immunogenic cell death induced by nanovaccine in tumor cells. (A) In vitro immunogenic cell death induction upon nanovaccine treatment in CT26 and B16F10 tumor cells. (B) Expression of HMGB1 and HSP90 after nanovaccine treatment or no treatment by western blotting in CT26 and B16F10 tumor cells.

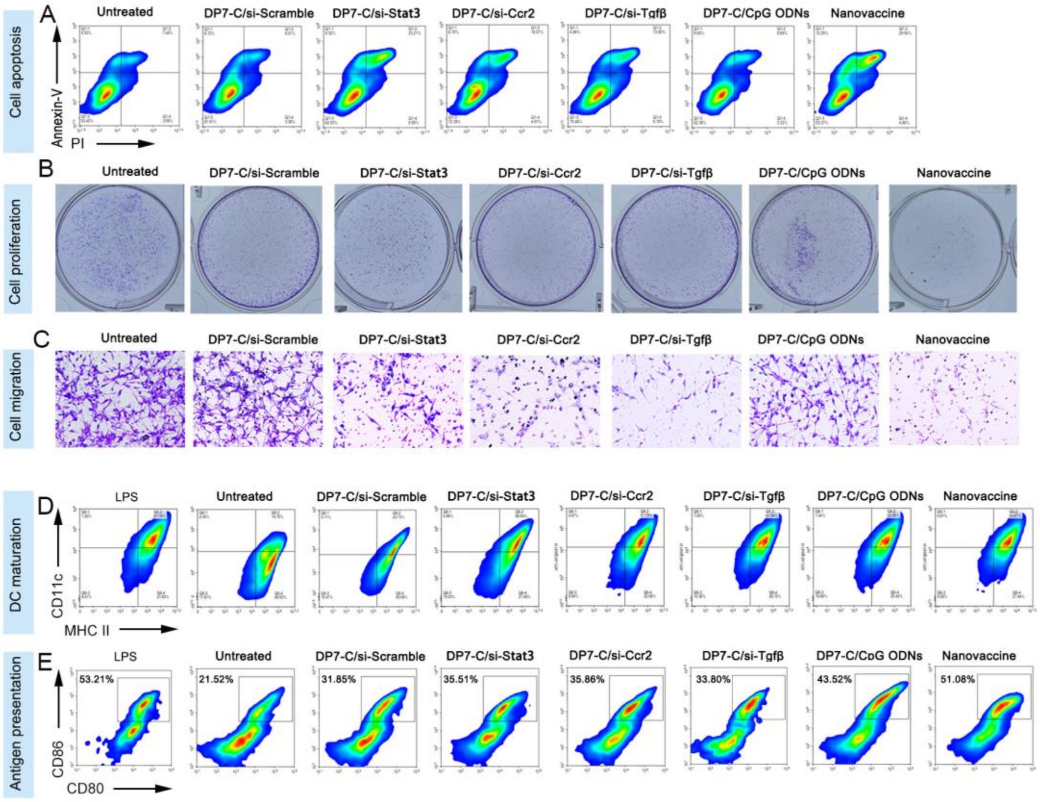

Figure S7. Anti-tumor response in vivo after treatment of nanovaccine. (A) Representative FACS images for cell apoptosis. (B) Representative images for clone formation assay. (C) Representative images for transwell migration analysis. (D,E) Representative FACS images for DC maturation.

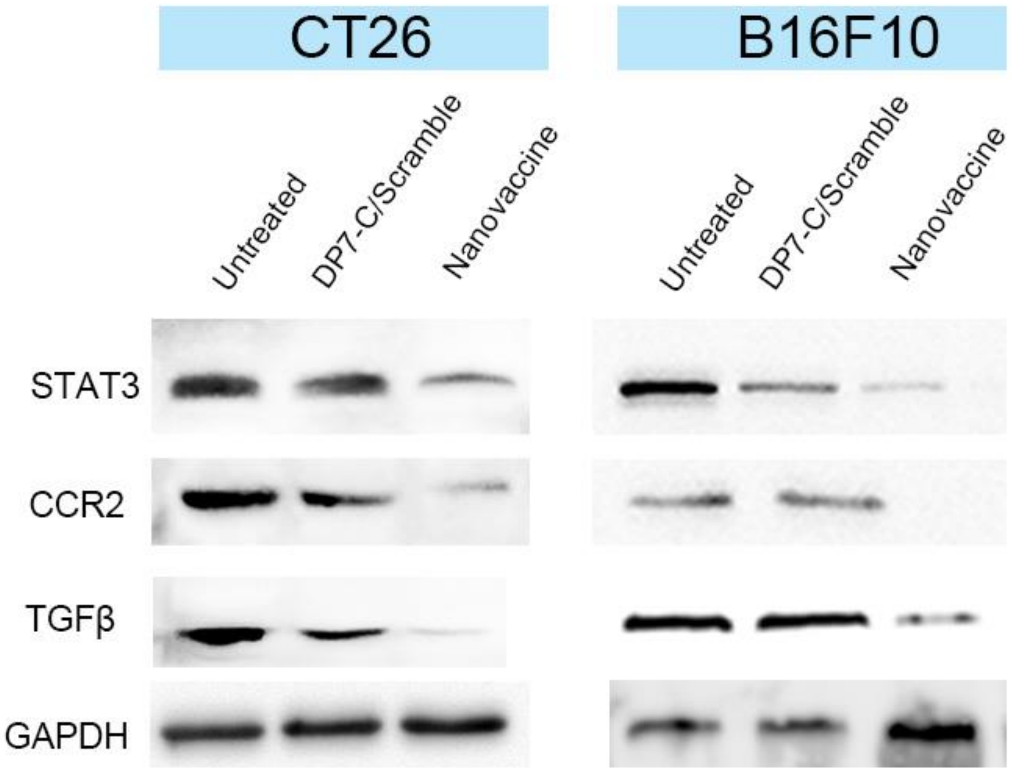

Figure S8. Target gene expression at the protein level detected by western blotting in tumor lysates.

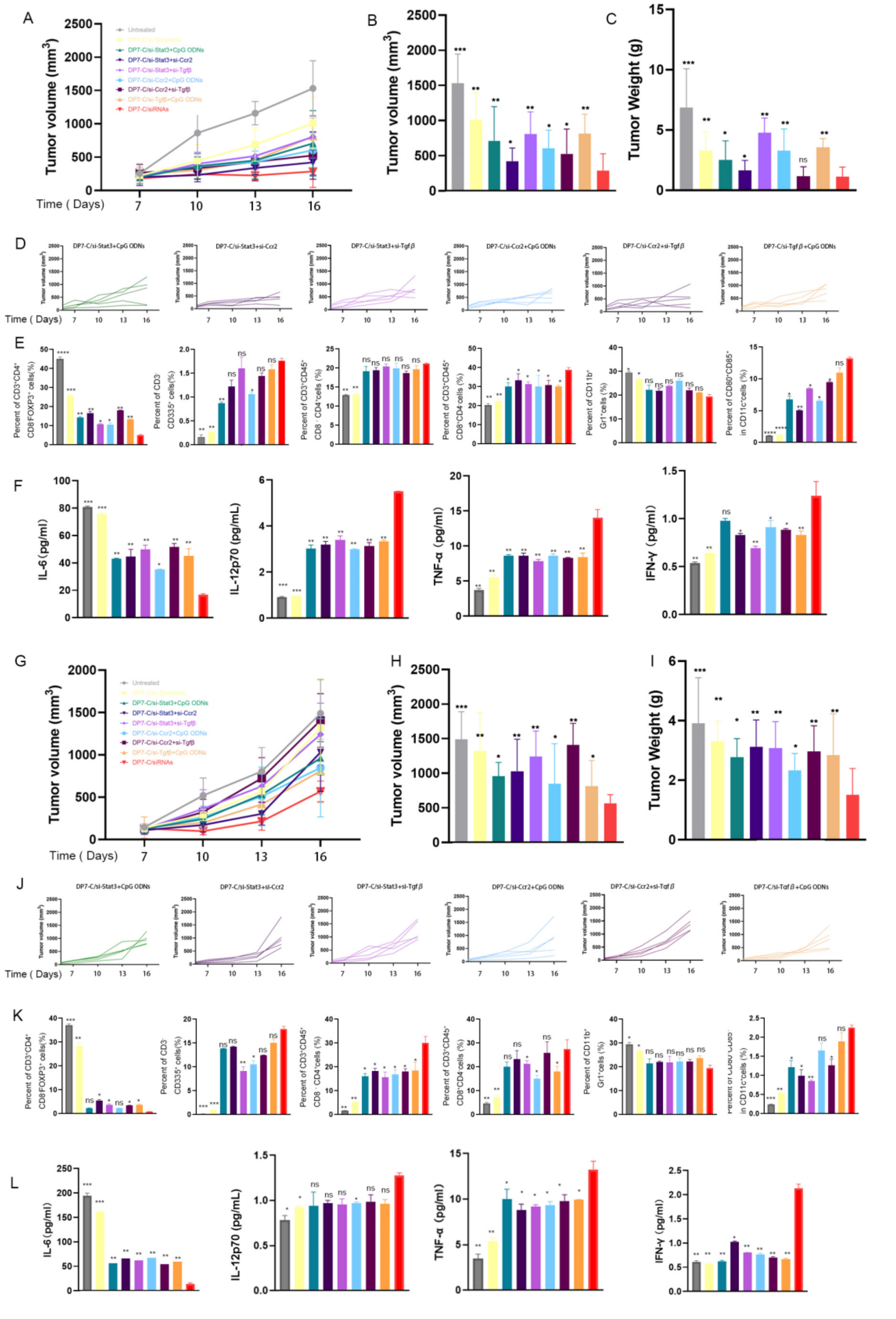

Figure S9. The nanovaccine effectively inhibited tumor growth. (A-D) Primary tumor volumes in CT26 models (A), mean tumor volumes on day 16 (B), tumor weights (C),

and tumor growth curves of the individual mice (D) in the CT26 model. (E) flow cytometry results of immune cells in CT26 tumors; (F) ELISA results of cytokines in CT26 tumors; (G-J) Primary tumor volumes (G), mean tumor volumes on day 16 (H), tumor weights (I), and tumor growth curves of the individual mice (J) in the B16F10 model. (K) flow cytometry results of immune cells in B16F10 tumors; (L) ELISA results of cytokines in B16F10 tumors.

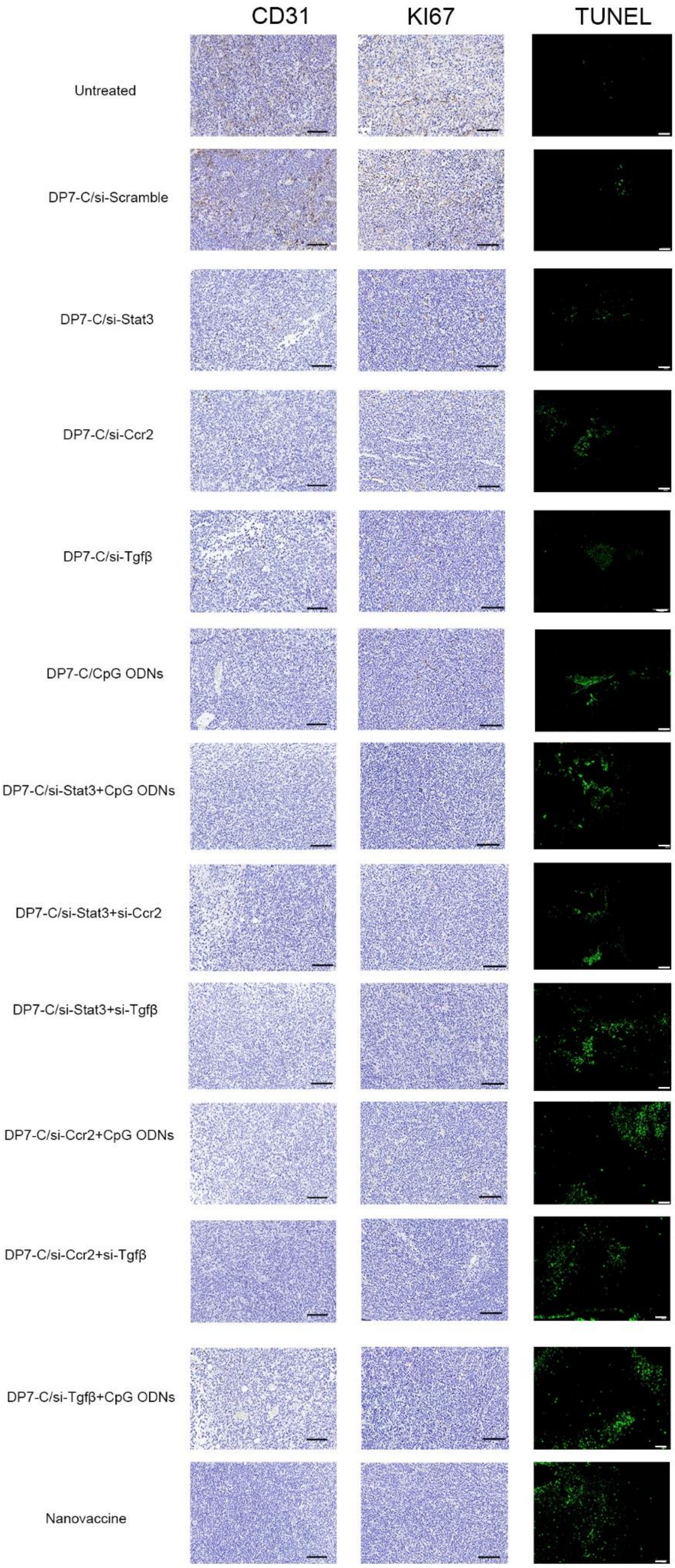
CD31
KI67
TUNEL
Untreated
DP7-C/si-Scramble
DP7-C/si-Stat3
DP7-C/si-Ccr2
DP7-C/si-Tgfβ
DP7-C/CpG ODNs
DP7-C/si-Stat3+CpG ODNs
DP7-C/si-Stat3+si-Ccr2
DP7-C/si-Stat3+si-Tgfβ
DP7-C/si-Ccr2+CpG ODNs
DP7-C/si-Ccr2+si-Tgfβ
DP7-C/si-Tgfβ+CpG ODNs
Nanovaccine

Figure S10. The nanovaccine repressed tumor angiogenesis and tumor cell proliferation. Immunohistochemistry staining of Ki67 and CD31as well as TUNEL staining.

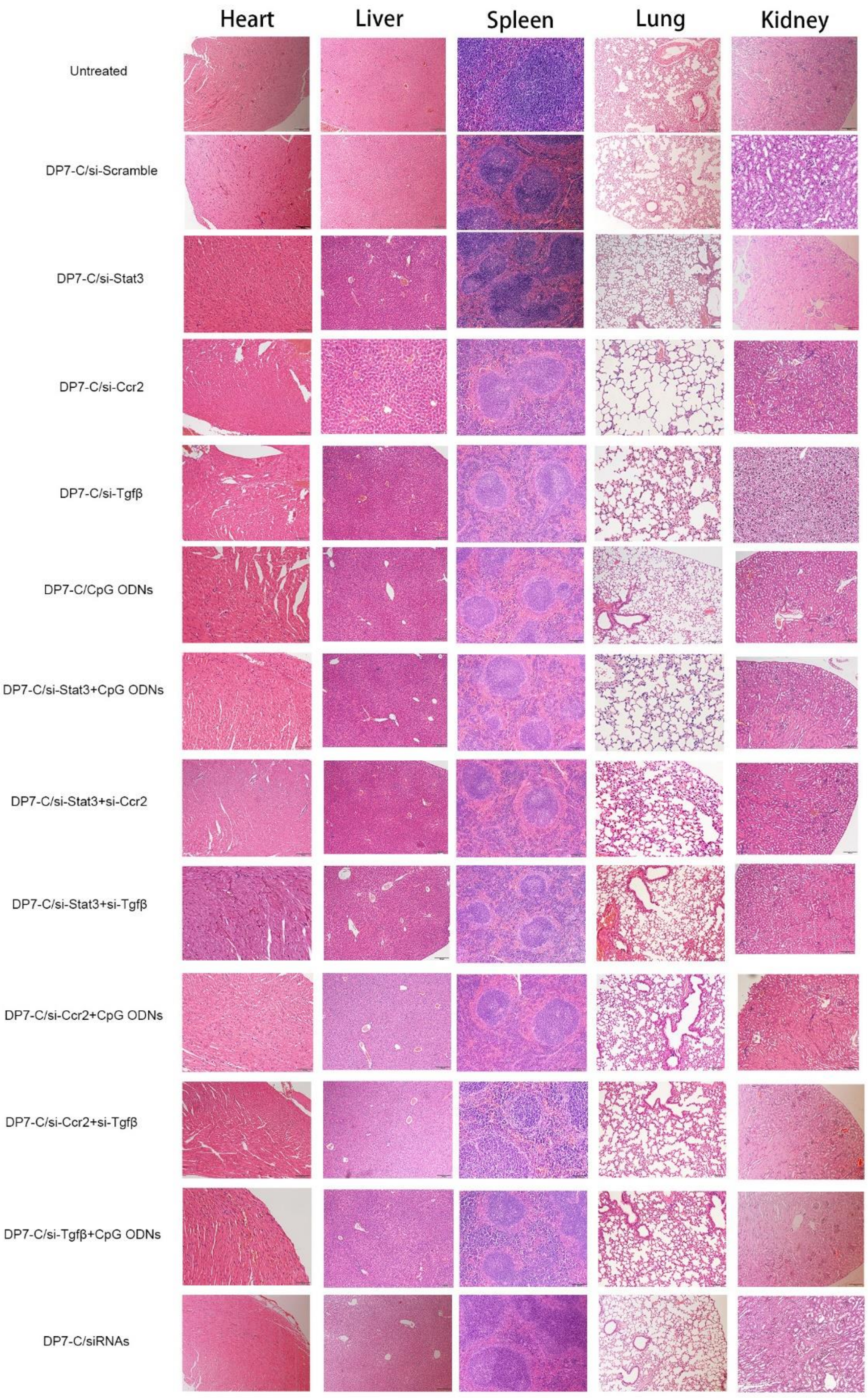

Figure S11. Safety and toxicity evaluation of the nanovaccine in vivo.

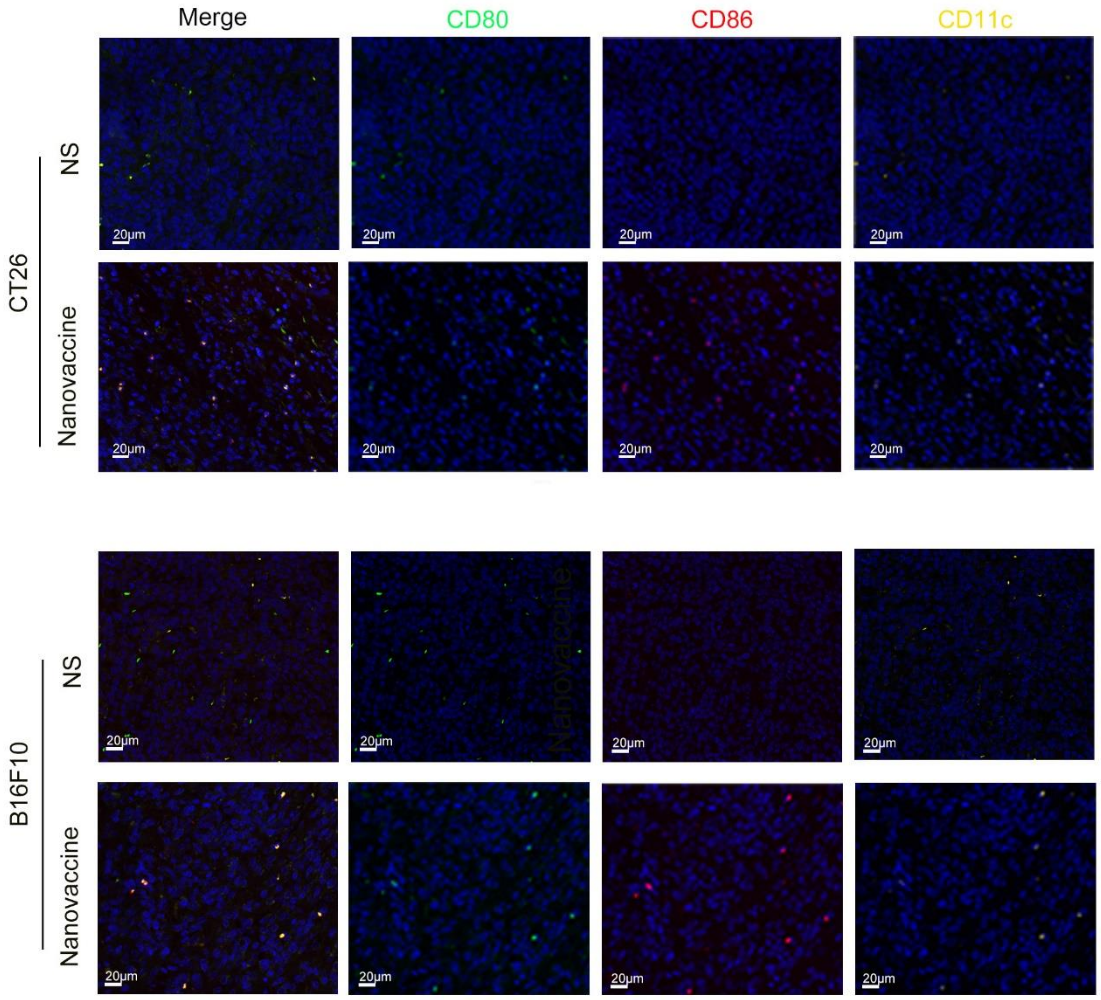

Figure S12. Immunofluorescent staining of CD80/CD86/CD11c DC cells in tumor sections at the end of the treatments.

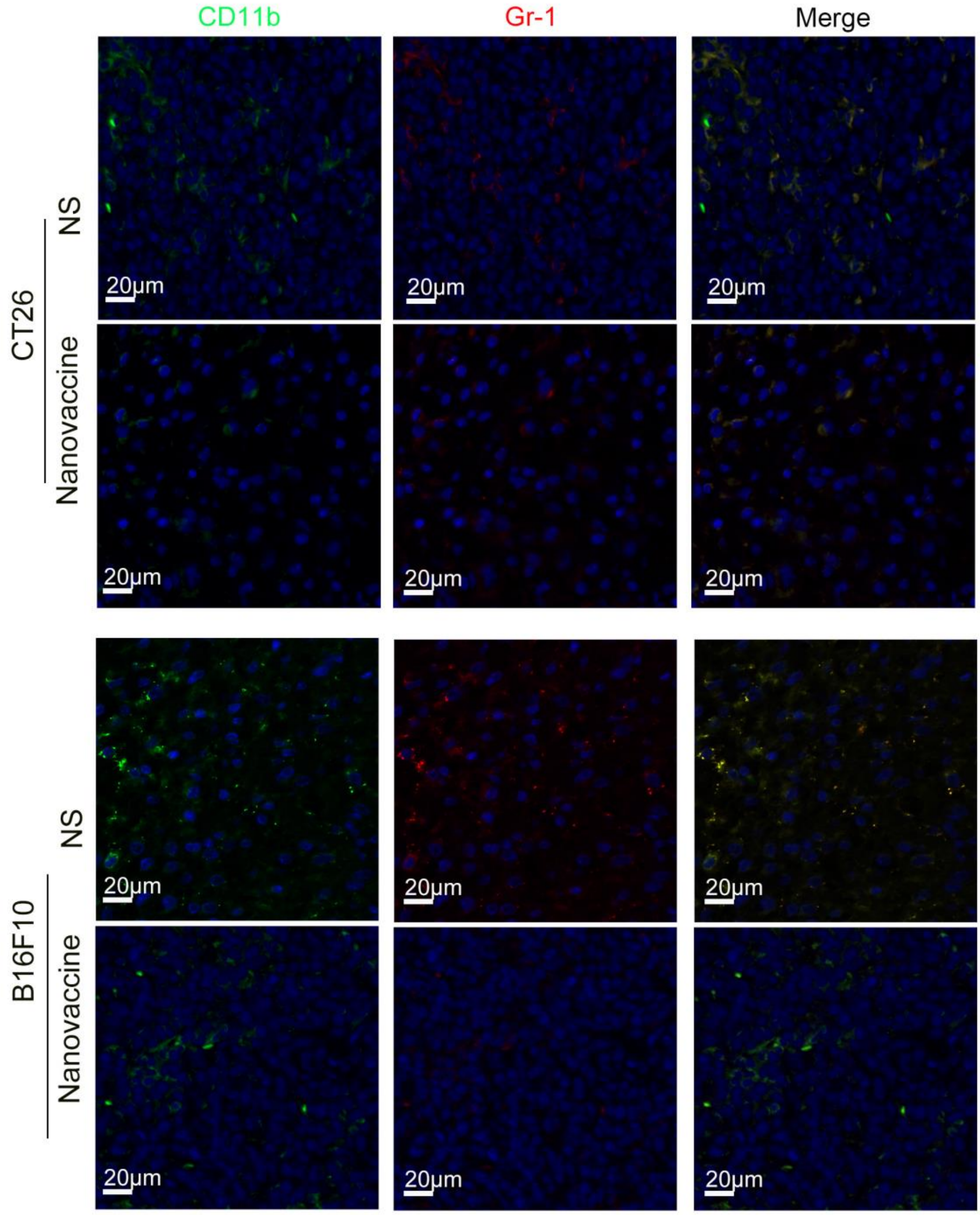

Figure S13. immunofluorescent staining of CD11b/Gr-1 MDSCs in tumor sections at the end of the treatments.

Supplementary table 1. Detailed target sequences of small nucleic acid

Name	Sense	Anti-sense
Stat3	5' - UUAGCCCAUGUGAUCUGACACCCUGAA -3'	5' - CAGGGUGUCAGAUCACAUGGGCUAA -3'
Ccr2	5' - GCAACAUGUUGGUCAUUAUTT -3'	5' - AUAAUGACCAACAUGUUGCTT -3'
Tgfβ	5' - CGGACUACUAUGCUAAAGATT -3'	5' - UCUUUAGCAUAGUAGUCCGTT -3'
Scramble	5'-UUCUCCGAACGUGUCACGUTT-3	5'-ACGUGACACGUUCGGAGAATT-3'
CpG ODNs	5' - TCCATGACGTTCCTGACGTT -3'	

Supplementary table 2. Overview of the primer sequences used in this study.

Primers	Sequences
Gapdh	F : 5' - GGTGAAGGTCGGTGTGAACG -3'<br>R : 5' - CTCGCTCCTGGAAGATGGTG -3
Stat3	F : 5' - TTCTCGTCCACCACCAAG -3'<br>R : 5' - GATATTGTCTAGCCAGACCC -3'
Ccr2	F : 5' - ACCTCAGTTCATCCACGGC -3'<br>R : 5' - ACAAGGCTCACCATCATCG -3
Tgfβ	F : CCAAGGAGACGGAATACAGG<br>R : GGGCTGATCCCGTTGATTTC

Supplementary table 3. Overview of the antibodies or dyes used in this study

Antibody	Source	Identifier
Anti-STAT3 (IHC, 1:100; WB, 1:1000)	CST	12640
Anti-CCR2 (IHC, 1:100; WB, 1:500)	Abcam	ab203128
Anti-TGFβ (IHC, 1:100; WB, 1:500)	Abcam	ab179695
Anti-human-CD4 (IF, 1:50)	CST	93518
Anti-human-CD8 (IF, 1:50)	CST	85336
Anti-HSP90 (IF/IHC, 1:50; WB, 1:1000)	Abcam	ab203085
Anti-HMGB1 (IF/IHC, 1:100; WB, 1:1000)	Abcam	ab18256
Anti-Calretinin (IF/IHC, 1:50)	Abcam	ab92341
Anti-CD8 (IF, 1:50)	CST	98941
Anti-FOXP3 (IF, 1:50)	CST	12653
Anti-PD-L1 (IF, 1:50; WB, 1:1000)	CST	60475
Anti-IL7R (IF, 1:50; WB, 1:1000)	Abcam	ab95024
Anti-BCL2 (IF, 1:50; WB, 1:500)	Proteintech	68103-1-Ig
Anti-CXCL9 (WB, 1:500)	Abcam	ab290643

Anti-CD4 (IF, 1:50; WB, 1:500)	CST	25229
Anti-IL12R (WB, 1:500)	Proteintech	13287-1-AP
Anti-CXCR2 (IF, 1:50; WB, 1:1000)	Abcam	ab65968
Anti-IRF1 (IF, 1:50; WB, 1:1000)	Abcam	ab232861
Anti-Caspase1 (IF, 1:50; WB, 1:1000)	Affinity	AF5418
Anti-NLRP3 (IF, 1:50; WB, 1:1000)	HUABIO	ET1610-93
Anti-STAT1 (IF, 1:50; WB, 1:1000)	Proteintech	10144-2-AP
Anti-VEGFA (IF, 1:50)	Abcam	EP1176Y
Anti-CD80 (IF, 1:50)	CST	54521
Anti-CD86 (IF, 1:50)	CST	19589
Anti-CD11c (IF, 1:50)	CST	97585
Anti-Gr-1 (IF, 1:50)	BD Biosciences	553125
Anti-Cd11b (IF, 1:50)	CST	17800
Anti-TNFα (IF, 1:50)	Abcam	ab183218
Anti-IL-1β (IF, 1:50)	Abcam	ab254360
Anti-Ki67 (IHC, 1:100)	Abcam	ab16667

Anti-CD31 (IHC, 1:100)	Abcam	ab182981
Anti-GAPDH (WB, 1:10000)	Abcam	ab8245
Alexa Fluor 488 (IF, 1:1000)	Thermo Fisher	A21206
Alexa Fluor 594 (IF, 1:1000)	Thermo Fisher	A11005
